# Extracellular Matrix of Echinoderms

**DOI:** 10.3390/md21070417

**Published:** 2023-07-22

**Authors:** Igor Yu. Dolmatov, Vladimir A. Nizhnichenko

**Affiliations:** A.V. Zhirmunsky National Scientific Center of Marine Biology, Far Eastern Branch, Russian Academy of Sciences, Palchevsky 17, 690041 Vladivostok, Russia

**Keywords:** echinoderms, extracellular matrix (ECM), mutable collagenous tissue, collagen, proteoglycan, glycoprotein, tensilin

## Abstract

This review considers available data on the composition of the extracellular matrix (ECM) in echinoderms. The connective tissue in these animals has a rather complex organization. It includes a wide range of structural ECM proteins, as well as various proteases and their inhibitors. Members of almost all major groups of collagens, various glycoproteins, and proteoglycans have been found in echinoderms. There are enzymes for the synthesis of structural proteins and their modification by polysaccharides. However, the ECM of echinoderms substantially differs from that of vertebrates by the lack of elastin, fibronectins, tenascins, and some other glycoproteins and proteoglycans. Echinoderms have a wide variety of proteinases, with serine, cysteine, aspartic, and metal peptidases identified among them. Their active centers have a typical structure and can break down various ECM molecules. Echinoderms are also distinguished by a wide range of proteinase inhibitors. The complex ECM structure and the variety of intermolecular interactions evidently explain the complexity of the mechanisms responsible for variations in the mechanical properties of connective tissue in echinoderms. These mechanisms probably depend not only on the number of cross-links between the molecules, but also on the composition of ECM and the properties of its proteins.

## 1. Introduction

The extracellular matrix (ECM), the most important innovation in the evolution of Metazoa, made it possible to form and maintain multicellularity [1]. In extant animals, connective tissue performs a wide variety of functions, from conducting cell–cell signals to creating support structures. In echinoderms, the ECM constitutes a substantial portion of tissue. Its composition, structure, and renewal play an important role in the physiology of these animals. The echinoderm connective tissue is capable of changing its mechanical properties. For this reason, it is referred to as mutable collagenous tissue (MCT) [2], or catch connective tissue [3]. Echinoderms use this ability for maintaining a posture (the catch state) [4,5], in case of autotomy [6,7], and in asexual reproduction [8,9,10,11]. Nevertheless, to date, the mechanisms changing the ECM strength and the substances involved are incompletely known [12,13].

The echinoderm connective tissue consists of proteins and polysaccharides, which are mostly homologous to those of other animals, especially vertebrates [14]. Its major part is composed of various types of collagens, glycoproteins, and proteoglycans. Although echinoderms and vertebrates have descended from a common ancestor and both belong to the Deuterostomia, they differ significantly in their connective tissue composition. In particular, echinoderms lack the *tropoelastin* gene and, accordingly, the ECM does not contain elastin. Unlike many other ECM proteins, elastin emerged within the vertebrate group and is absent from agnathans and lower chordates, as well as from invertebrates [15]. An assumption has been made that the *tropoelastin* gene was formed on the basis of the *fibrillin* gene [16].

One of the major mechanisms of origin and evolution of connective tissue proteins is the domain shuffling of pre-existing domains [1]. In this regard, identifying ECM proteins of non-model species often poses a challenge, since their domain composition may differ from the “typical” one. Examples of such proteins are tenascins and fibronectins. These play an important role in the structural integrity of ECM in vertebrates [17,18]. Echinoderms have proteins that contain domains characteristic of tenascin and fibronectin such as FBG, EGF, TILa, and FN3 [19,20]. However, all these domains are ancient in origin and are found in a variety of animals. As a combination typical of tenascins and fibronectins, these are observed only in chordates [1,21].

ECM components are undoubtedly involved in the mechanisms changing the MCT properties. In this regard, addressing the question as to how the echinoderm ECM and its associated “adhesome” has evolved as a system and what the differences are from the vertebrate ECM is important for understanding its normal functions and mechanisms responsible for changing the mechanical properties of connective tissue. In this review, which incorporates our own previously unpublished analysis (for methods, see Section 5), we attempted to identify the major proteins that constitute connective tissue and can potentially be involved in the mechanisms changing its mechanical properties in echinoderms.

## 2. Structural Components of Connective Tissue

### 2.1. Collagens

Collagens are a superfamily of proteins that are the key structural components of ECM. In vertebrates, this superfamily comprises 28 members [22]. Collagens are homo- or heterotrimers consisting of the so-called collagen alpha chains. Humans have 44 genes encoding alpha chains [22]. The major characteristic feature of collagens structure is the presence of a collagen domain containing triple-helix (Gly-X-Y) motifs, where X is most frequently proline, and Y is hydroxyproline [23]. The number and organization of these motives varies greatly between different members of the family. Collagens are also distinguished by the presence of various domains that impart various properties to them. The most common domains are the N-terminal signaling peptide, thrombospondin (THBS) domain, von Willebrand factor type A domain (vWA), and Fibronectin type-3 repeat (FB3). The THBS domain performs a regulatory function, while vWA and FB3 are responsible for binding to other proteins and adhesion [24]. To date, six groups of collagens have been identified in mammals: fibril-forming collagens, fibril-associated collagens with interrupted triple helices (FACITs), network-forming collagens, collagens VI, VII, XXVI, and XXVIII, membrane collagens and multiplexins [22]. Collagens of all groups have been found in echinoderms except for membrane collagens.

#### 2.1.1. Fibril-Forming Collagens

**Figure 1:**
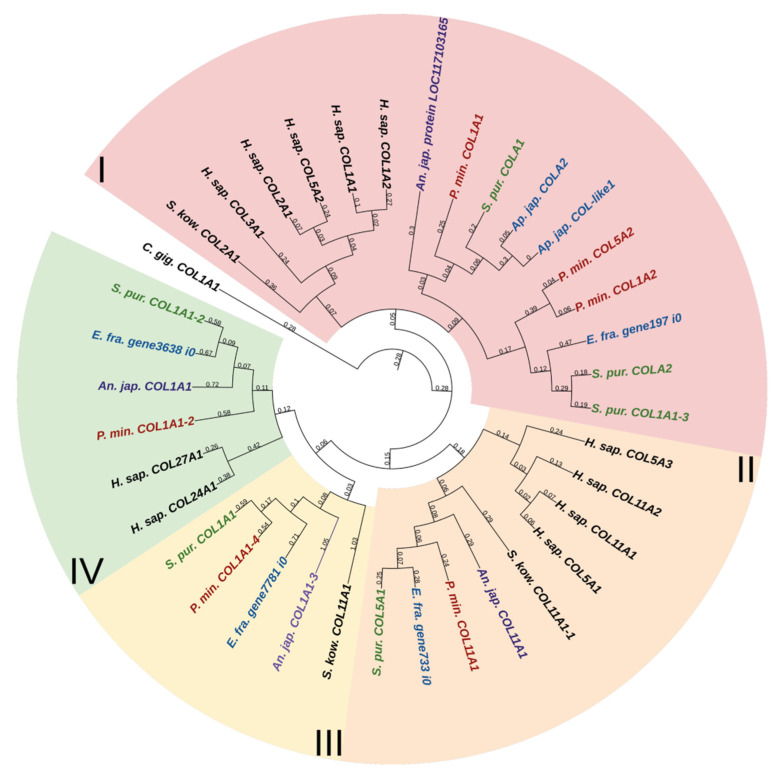
Phylogenetic tree showing the relationships of fibril-forming collagens of vertebrates, hemichordates, and echinoderms. Crinoids (*Anneissia japonica*)—purple color; asteroids (*Patiria miniata*)—red color; echinoids (*Strongylocentrotus purpuratus*)—green color; holothurians (*Apostichopus japonicus*/*Eupentacta fraudatrix*)—blue color; hemichordates (*Saccoglossus kowalevskii*); vertebrates (*Homo sapiens*)—black color. Groups of proteins (I–IV) are marked with colored areas.

Fibril-forming collagens, or fibrillar collagens, are the most widespread type of collagens. These include collagens I, II, III, V, XI, XXIV, and XXVII. Such collagens perform a structural function in all animals. Echinoderms have from four to six proteins of this type. In the constructed phylogenetic tree of fibrillar collagens, four protein groups can be distinguished (Figure 1, Supplementary File S1). The first includes two subgroups: one is formed by collagens of echinoderms, the other by collagens I, II, III, VA2 of vertebrates and hemichordates. This division probably indicates a significant divergence of these collagens in echinoderms after the separation of Ambulacraria.

The second group comprises homologues of type V and XI collagens. This group of echinoderm proteins can be designated as collagens V/XI. The third one contains sequences found only in members of Ambulacraria. No homology with any types of fibril-forming collagens of vertebrates can be found for them due to the significant divergence and/or loss of the ancestral gene by chordates. The fourth group contains homologues of human collagens XXIV and XXVII. Thus, the phylogenetic analysis indicates that homologs of all types of fibrillar collagens characteristic of vertebrates are present in echinoderms.

**Figure 2:**
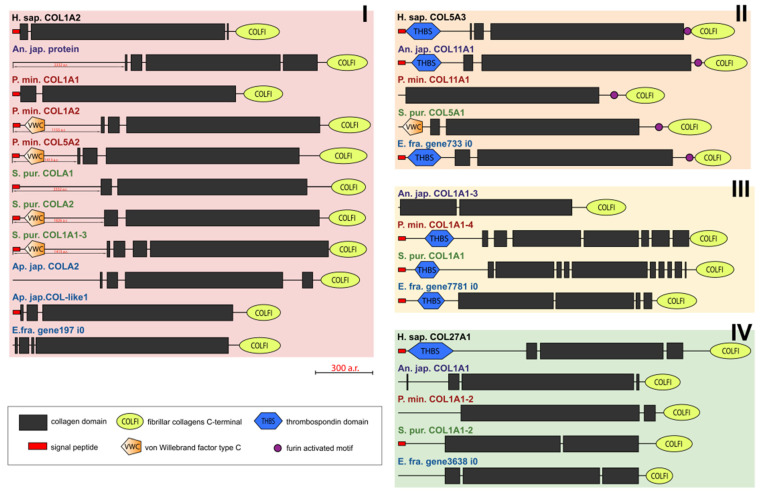
Domain structure of fibril-forming collagens of echinoderms. The length sizes of proteins and domains in the image reflect the lengths of their amino acid sequences. Crinoids (*Anneissia japonica*)—purple color; asteroids (*Patiria miniata*)—red color; echinoids (*Strongylocentrotus purpuratus*)—green color; holothurians (*Apostichopus japonicus*/*Eupentacta fraudatrix*)—blue color; hemichordates (*Saccoglossus kowalevskii*); vertebrates (*Homo sapiens*)—black color. Groups of proteins (I-IV) are marked with colored areas.

The molecules of fibril-forming collagens of vertebrates and echinoderms contain one main triple-helical domain and a C-terminal propeptide (Figure 2). Many fibrillar collagens bear the THBS domain at the N-terminus [25]. As in COL5A3 of *H. sapiens*, in some fibrillar collagens of echinoderms, the furin-activated motif (R-X-R/K-R) is located at the boundary of the triple-helical site and C-terminal propeptide. It suggests that the propeptide can be removed using furin [26].

Some fibrillar collagens of the sea urchin *S. purpuratus* and the sea star *P. miniata* contain the N-terminal domain von Willebrand factor type C (vWC) which is found neither in vertebrates’ fibrillar collagens nor in other collagens of echinoderms. Although vWA, a similar domain, is characteristic of the groups FACITs and collagens VI, VII, XXVI, and XXVIII of vertebrates, the sequences bearing it are identified by BLASTp as fibrillar collagens and contain the C-terminal propeptide of this group.

An analysis of the domain structure and phylogenesis of fibrillar collagens shows that the genes of echinoderms of group I are homologous to the genes encoding vertebrate collagens I, II, and III. Apparently, they arose on the basis of one ancestral gene as a result of its duplication and divergence. At the same time, proteins with the vWC domain are probably a late acquisition of echinoderms and were formed after the separation of Asterozoa and Echinozoa. These collagens form a separate subgroup. It includes the E. fra protein197 i0. Since it was not the genome that was used but the transcriptome of *E. fraudatrix*, it is possible that the transcript of Efra.gene197_i0 is incomplete. In this regard, echinoderm proteins belonging to group I can be referred to as collagens I/II/III.

#### 2.1.2. FACITs

**Figure 3:**
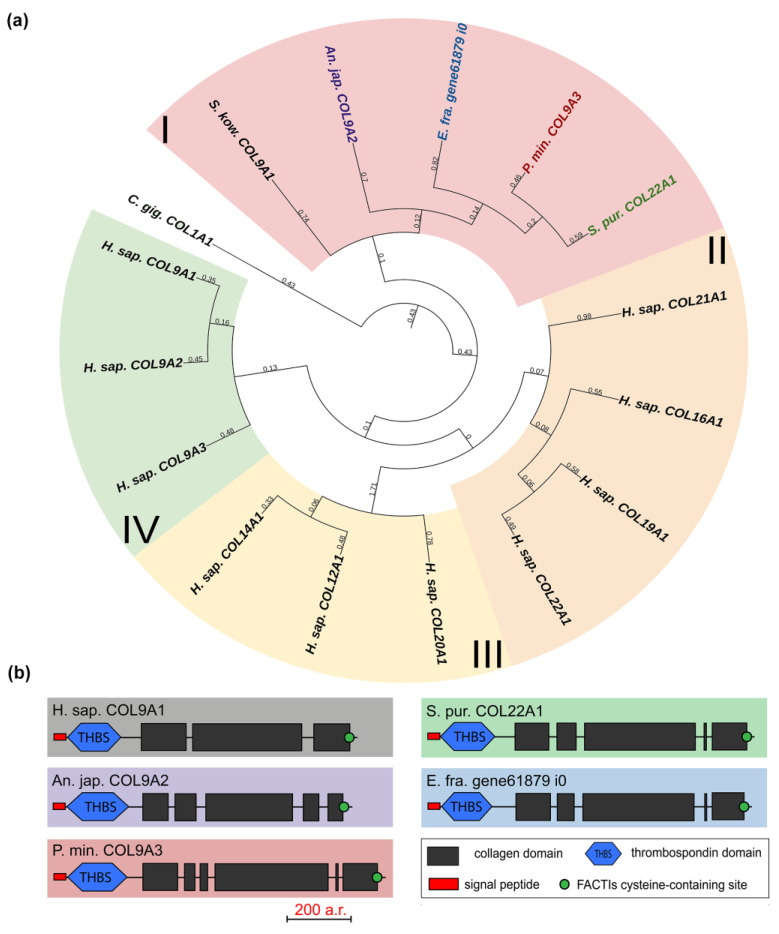
FACITs of echinoderms. (**a**). Phylogenetic tree showing the relationships of FACITs of vertebrates, hemichordates, and echinoderms. Groups of proteins (I–IV) are marked with colored areas. (**b**). Domain structure of FACITs of echinoderms. The length sizes of proteins and domains in the image reflect the lengths of their amino acid sequences. Crinoids (*Anneissia japonica*)—purple color; asteroids (*Patiria miniata*)—red color; echinoids (*Strongylocentrotus purpuratus*)—green color; holothurians (*Eupentacta fraudatrix*)—blue color; hemichordates (*Saccoglossus kowalevskii*); vertebrates (*Homo sapiens*)—black color.

**Figure 4:**
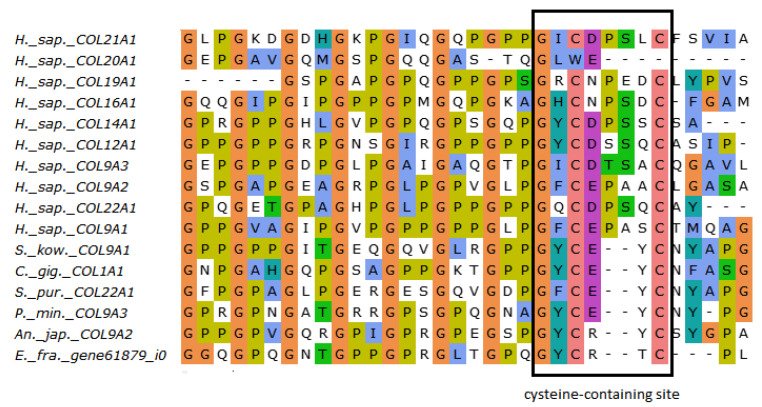
MUSCLE (Ugene) alignment of amino acid sequences of FACITs cysteine-containing sites of vertebrates, hemichordates, and echinoderms.

The FACITs group comprises collagens IX, XII, XIV, XVI, XIX, XX, XXI, and XXII. In the studied echinoderm species, one FACITs gene was found. In the phylogenetic tree, they form a separate group, which indicates a significant divergence of FACITs of deuterostomes (Figure 3a, Supplementary File S2). The major structural feature of FACITs is the presence of several short triple-helical domains flanked by non-collagenous sites, which imparts flexibility to the molecule (Figure 3b). Most vertebrate FACITs contain vWA and THBS domains at the N-terminus, while some have FB3 repeats separating these structures [27]. In the echinoderm species studied, FACITs contain only the THBS domain and are closest in structure to collagens IX of *H. sapiens*. In this regard, this group of echinoderm collagens can be conventionally referred to as collagens IX. A characteristic feature of FACITs is the cysteine-containing site GXCXXXXC at the C-terminus [28]. This site is required for trimerization of polypeptide chains [28]. Echinoderm FACITs have been found to have a similar site (GXCXXC) in this region, which is aligned with human FACITs (Figure 4, Supplementary File S2). FACITs do not form fibrils but are associated with fibrillar collagens and other proteins, acting as a link between different ECM components [27]. In this regard, echinoderm collagens IX may be involved in the processes changing the mechanical properties of MCT.

#### 2.1.3. Network-Forming Collagens

**Figure 5:**
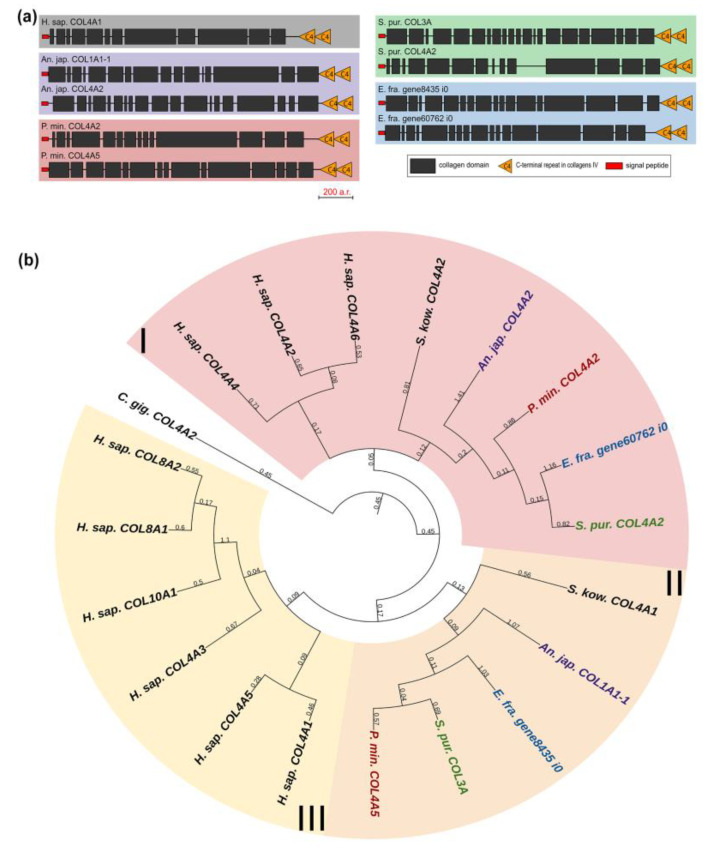
Network-forming collagens of echinoderms. (**a**). Domain structure. The length sizes of proteins and domains in the image reflect the lengths of their amino acid sequences. (**b**). Phylogenetic tree showing the relationships of network-forming collagens of vertebrates, hemichordates, and echinoderms. Crinoids (*Anneissia japonica*)—purple color; asteroids (*Patiria miniata*)—red color; echinoids (*Strongylocentrotus purpuratus*)—green color; holothurians (*Eupentacta fraudatrix*)—blue color; hemichordates (*Saccoglossus kowalevskii*); vertebrates (*Homo sapiens*)—black color. Groups of proteins (I–III) are marked with colored areas.

Collagens IV, VIII, and X form a group of network-forming collagens. The former two support the structure of basement membranes, while collagen X is presumably involved in bone mineralization during the embryonic development of vertebrates [29]. Collagen IV is distinguished by the length and organization of the alpha chain that has a large number of interruptions. Network-forming collagens also differ in C-terminal sites: collagens X and VIII end with the C1q domain, which is necessary for proper folding and protein–protein interactions [30]; collagens IV end with the duplicated C4 domain, an important component of basement membranes [31]. Echinoderms have two genes from this group, and, judging by the presence of a C-terminal duplicated domain in all of them, these are homologs of collagens IV (Figure 5a). This is also confirmed by the phylogenetic analysis (Figure 5b, Supplementary File S3).

Collagen IV is a fundamental component of basement membranes. The latter are ancient and highly conserved forms of ECM [32]. Therefore, the main functions of their components in different animal phyla, including echinoderms, should be similar. It is worth mentioning that a number of genes encoding proteins of the basic set of “basement membrane ECM toolkit” (collagens IV and XV/XVIII, laminin, nidogen, and perlecan) shared by protostomes and deuterostomes [14], increase their expression in the body wall of *Cladolabes schmeltzii* during fission of individuals, which indicates a substantial role of the components of this structure in the processes of connective tissue rearrangement [20].

#### 2.1.4. Collagens VI, VII, XXVI, and XXVIII

**Figure 6:**
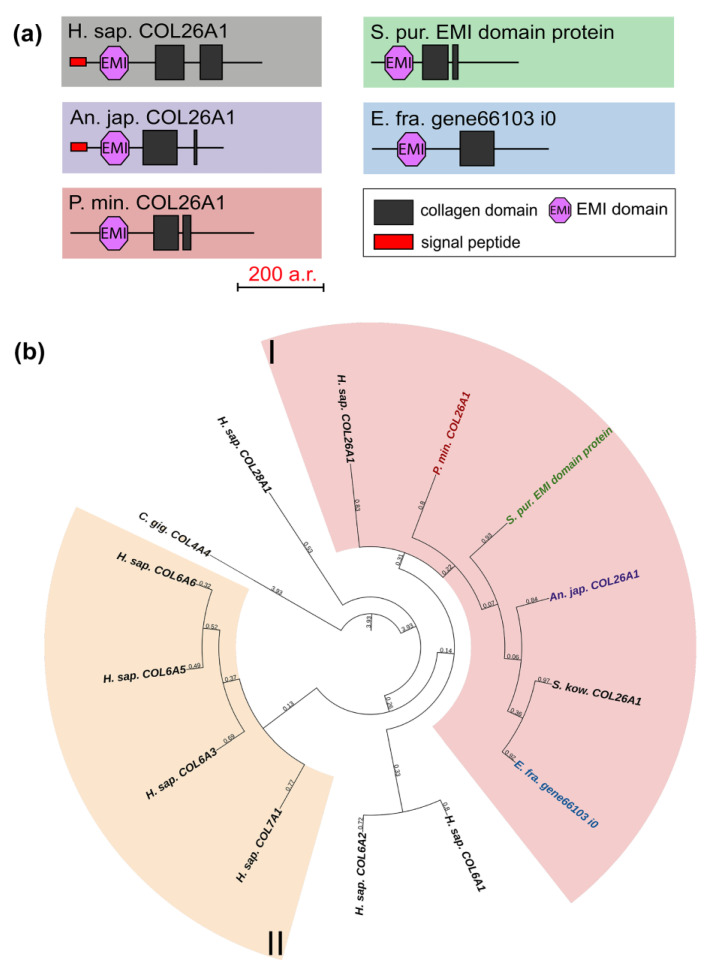
Collagens VI, VII, XXVI, and XXVIII of echinoderms. (**a**). Domain structure. The length sizes of proteins and domains in the image reflect the lengths of their amino acid sequences. (**b**). Phylogenetic tree showing the relationships of collagens VI, VII, XXVI, and XXVIII of vertebrates, hemichordates, and echinoderms. Crinoids (*Anneissia japonica*)—purple color; asteroids (*Patiria miniata*)—red color; echinoids (*Strongylocentrotus purpuratus*)—green color; holothurians (*Eupentacta fraudatrix*)—blue color; hemichordates (*Saccoglossus kowalevskii*); vertebrates (*Homo sapiens*)—black color. Groups of proteins (I, II) are marked with colored areas.

Collagens VI, VII, XXVI, and XXVIII are important components of basement membranes and microfibrillar networks [33,34,35]. The domain organization of these collagens is similar between vertebrates: besides the collagen domain, all have vWA, and some have fibronectin repeats and the C-terminal Kunitz domain, which is cut off during protein maturation [22]. The exception is collagen XXVI, which contains only two triple-helix sites and the EMI domain [36]. In this group, only one gene was found in echinoderms. Its products contain one EMI and one or two triple-helix domains (Figure 6a). In the phylogenetic tree, these are grouped with human collagen XXVI, in connection with which they can be referred to as collagens XXVI (Figure 6b, Supplementary File S4). The function of collagen XXVI remains unclear. In vertebrates, it is known to be expressed in testes and ovaries and is presumably involved in the development of these organs [37]. The presence of the EMI domain required for the multimerization of proteins indicates the ability of collagen XXVI to bind to other ECM components that contain such domains as laminin-type EGF-like, collagen-like, etc. [38].

#### 2.1.5. Multiplexins

**Figure 7:**
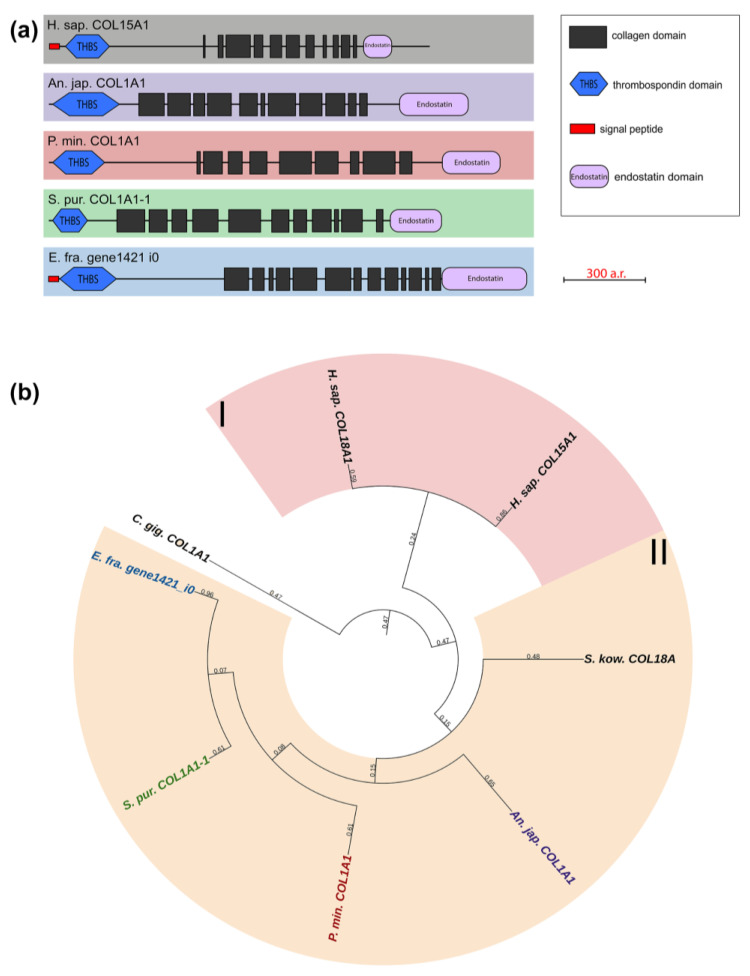
Multiplexins of echinoderms. (**a**). Domain structure. The length sizes of proteins and domains in the image reflect the lengths of their amino acid sequences. (**b**). Phylogenetic tree showing the relationships of Multiplexins of vertebrates, hemichordates, and echinoderms. Crinoids (*Anneissia japonica*)—purple color; asteroids (*Patiria miniata*)—red color; echinoids (*Strongylocentrotus purpuratus*)—green color; holothurians (*Eupentacta fraudatrix*)—blue color; hemichordates (*Saccoglossus kowalevskii*); vertebrates (*Homo sapiens*)—black color. Groups of proteins (I, II) are marked with colored areas.

Multiplexins are a special group of collagens that includes two proteins: collagens XV and XVIII. These have sequences for attaching heparan sulfate and chondroitin sulfate side chains and are proteoglycans by their structure [39,40]. Their molecules consist of 9–11 intermittent triple-helix sites, a signal peptide, and the THBS domain at the N-terminus and the Endostatin domain at the C-terminus. The echinoderms that we studied have one multiplexin gene with a similar structure. Most of them are referred in the NCBI database to as Collagen type I. A BLASTp analysis showed the best hits among network-forming and fibrillar collagens. However, their domain organization is close to vertebrate multiplexins (Figure 7, Supplementary File S5). It would probably be more correct to refer to collagens of this group in echinoderms as collagens XV/XVIII.

Collagens XV and XVIII are important components of basement membrane required for maintaining its integrity [39]. Due its specific structure, their molecule has a complex “knot/figure-of-eight/pretzel” configuration, which allows forming links with other connective-tissue fibrils [40]. In addition to the structural function, these collagens are involved in the development and carcinogenesis of many tissues [41,42]. For instance, the Endostatin domain is capable of inhibiting migration, proliferation, and causing apoptosis of endothelial cells [43,44]. The possible involvement of multiplexins in the transformation of the body-wall ECM has been reported for holothurians. Expression of collagen XV/XVIII increases in case of asexual reproduction in *C. schmeltzii* [20]. 

#### 2.1.6. Unknown Collagens

**Figure 8:**
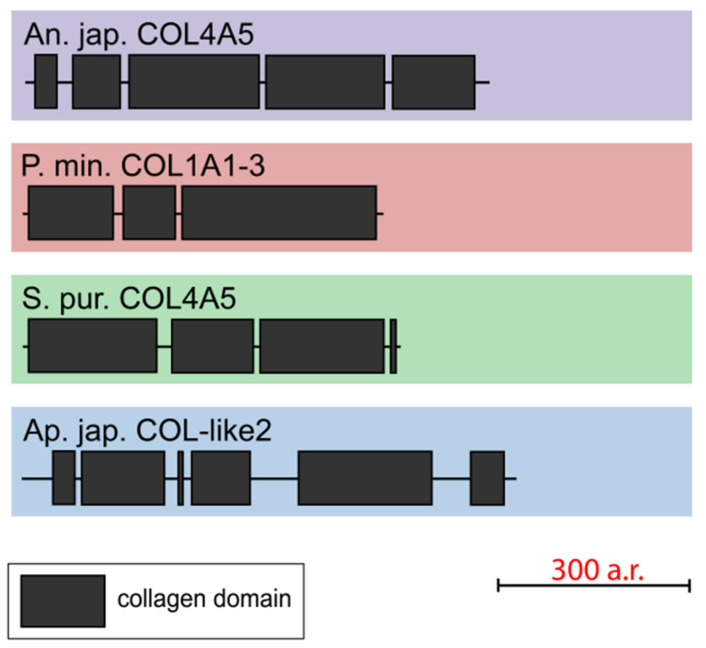
Domain structure of echinoderm unknown collagens. The length sizes of proteins and domains in the image reflect the lengths of their amino acid sequences.

One gene that encodes collagen-like short-length (563–753 aa) proteins was found in each of all the studied echinoderms. The NCBI database contains homologous sequences of other echinoderms with similar lengths and structures. All the found proteins consist of three to six discontinuous triple-helical domains (Figure 8, Supplementary File S6). These collagens mostly resemble FACITs in the number of such sites and their length. However, these proteins lack the THBS domain and cysteine-containing site characteristic of FACITs. Thus, echinoderms have collagens that are clearly different from those of vertebrates and do not fit into any of the groups. It is likely that these proteins are an ancestral form of deuterostome collagens or evolved in echinoderms on the basis of FACITs or fibrillar collagens through a loss of some domains.

### 2.2. Proteoglycans

Proteoglycans and glycoproteins are multifunctional components of connective tissue which mediate adhesion, proliferation, differentiation, and migration of various cells [45,46,47]. The difference between them is relatively arbitrary. In this review, we differentiate them according to Hynes and Naba [48]. The presence of proteoglycans in MCT has been shown for different classes of echinoderms [49]. Presumably, these, along with stiffening proteins like tensilin and NSF, are a link between neighboring collagen fibrils and are involved in MCT transformation [50]. Our analysis showed that echinoderms lack genes of a number of proteoglycans characteristic of vertebrates such as aggrecan, versican, brevican, neurocan, and decorin.

#### 2.2.1. Syndecans

**Figure 9:**
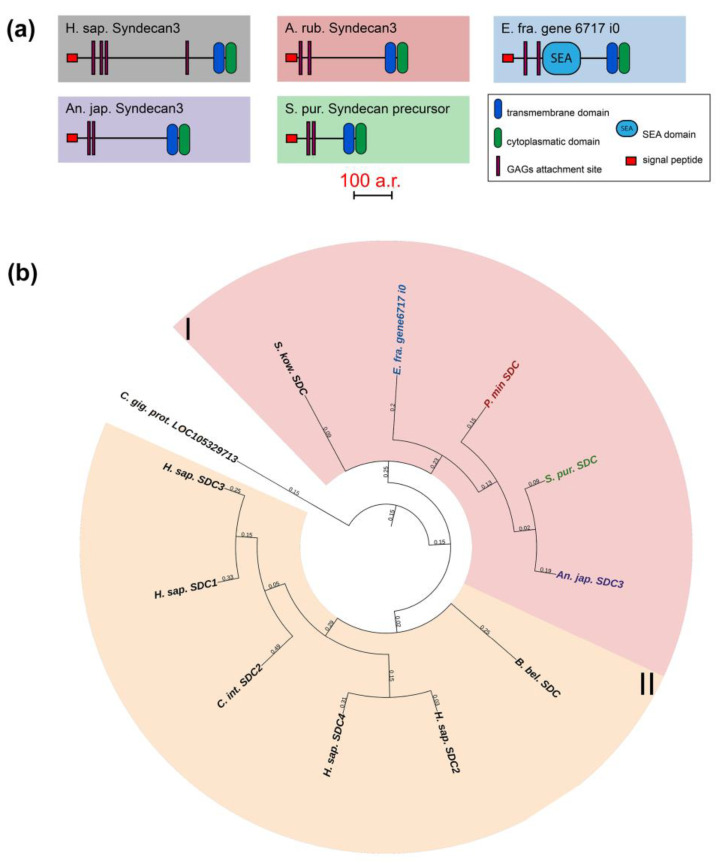
Syndecans of echinoderms. (**a**). Domain structure. The length sizes of proteins and domains in the image reflect the lengths of their amino acid sequences. (**b**). Phylogenetic tree showing the relationships of Syndecans of chordates, hemichordates, and echinoderms. Crinoids (*Anneissia japonica*)—purple color; asteroids (*Patiria miniata*)—red color; echinoids (*Strongylocentrotus purpuratus*)—green color; holothurians (*Eupentacta fraudatrix*)—blue color; hemichordates (*Saccoglossus kowalevskii*) and chordates (*Ciona intestinalis*, *Branchiostoma belcheri* and *Homo sapiens*)—black color. Groups of proteins (I, II) are marked with colored areas.

The syndecan (SDC) family includes transmembrane proteoglycans that form homodimers and perform function of co-receptors of cell surface proteins such as integrins [51]. Mammals have four genes of this family, while only one has been found in echinoderms (Figure 9, Supplementary File S7). The domain structures of echinoderm and mammalian syndecans are similar and include a signal peptide, an extracellular domain represented by glycine-serine motifs and the transmembrane and C-terminal cytoplasmic domains [52]. The exception is the presence of the SEA domain in the central region of the syndecans in some echinoderms. It is reliably detected by NCBI CD Search and SMART in the holothurians *E. fraudatrix* and *A. japonicus*. SMART also identifies SEA in *H. leucospilota* and the sea stars *A. rubens* and *P. miniata*, but below the probability threshold. 

The extracellular domain can bind various glycosaminoglycans (GAGs), which determine the function of entire molecule, by means of glycine-serine motifs [53,54,55]. Depending on the type of GAGs, syndecan can interact with growth factors, cytokines, enzymes, and other ECM molecules [52]. The transmembrane domain is required for the formation of homodimers by syndecans. The cytoplasmic domain is involved in intracellular interactions. By means of two conservative sites (C1 and C2), it can bind both to kinases for signal transmission and directly to cytoskeletal proteins [52]. Thus, syndecan is potentially capable of regulating many processes in echinoderms and may mediate various ECM rearrangements that occur with changes in the MCT state. In sea urchin embryos, it is involved in post-oral arm formation due to its effect on cell proliferation [35]. During the asexual reproduction in the holothurian *C. schmeltzii*, the *syndecan* expression decreases, which may indicate its probable involvement in fission and transformation of the body-wall connective tissue [20].

#### 2.2.2. Glypicans

**Figure 10:**
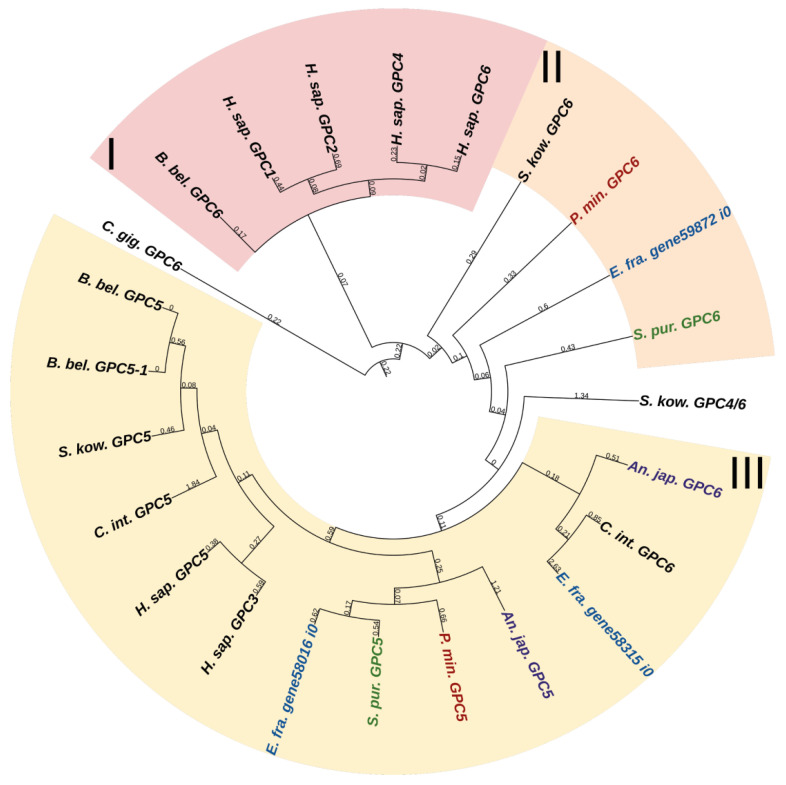
Phylogenetic tree showing the relationships of Glypicans of chordates, hemichordates, and echinoderms. Crinoids (*Anneissia japonica*)—purple color; asteroids (*Patiria miniata*)—red color; echinoids (*Strongylocentrotus purpuratus*)—green color; holothurians (*Eupentacta fraudatrix*)—blue color; hemichordates (*Saccoglossus kowalevskii*) and chordates (*Ciona intestinalis*, *Branchiostoma belcheri* and *Homo sapiens*)—black color. Groups of proteins (I–III) are marked with colored areas.

Glypicans (GPC) are a family of heparan sulfate proteoglycans located on the outer surface of plasma membrane [56]. These proteoglycans are divided into two subfamilies. In mammals, the first subfamily is glypicans 1, 2, 4, and 6, and the second one is glypicans 3 and 5 [56] (Figure 10, Supplementary File S8). Crinoids, asteroids, ophiuroids, and echinoids have one glypican gene in each of the subfamilies (Figure 10). Holothurians have two genes in the first subfamily and one gene in the second subfamily. Glypicans consist of heparan sulfate chains, a core protein, and glycosylphosphatidylinositol (GPI) linkage required for attachment to the cell membrane [57]. The location on the cell surface allows these proteins to interact with extracellular components of various signaling pathways. Thus, vertebrate glypicans, regulating such signaling pathways as Wnt, Hedgehog (Hh), fibroblast growth factor (FGF), bone morphogenetic protein (BMP), and Hippo are involved in various development and carcinogenesis processes [56,58]. In the sea urchin *Paracentrotus lividus*, glypican 5 is regulated by BMP signaling and shows a variable pattern of expression at the blastula and gastrula stages [59]. Due to the large repertoire of regulated signaling pathways, glypicans can have an effect on ECM remodeling and on the MCT properties change.

#### 2.2.3. Betaglycan

Betaglycan, or Transforming growth factor beta receptor III (TGFBR3), is a heparan sulfate proteoglycan of cell surface consisting of a large extracellular, transmembrane, and short cytoplasmic domains [60]. It is a co-receptor for some ligands of the TGF-β signaling pathway which is involved in many processes including embryonic development, cell differentiation, proliferation, immune system control, and regeneration [61]. TGFBR3 can also acquire a soluble form. Due to its proteolytic cleavage, or shedding, by means of matrix metalloproteinase MT1-MMP, ectodomain is released into the extracellular space [62]. The free ectodomain binds to ligands and becomes involved in their deposition and neutralization [63]. Thus, the membrane and soluble forms of betaglycan can perform opposite functions. Each echinoderm has one TGFBR3 gene with a similar structure. There is a lack of information about the betaglycan functions in echinoderms. We assume that, as a participant in TGF-β signaling, it can potentially be involved in ECM remodeling.

#### 2.2.4. Bamacan

Bamacan, or structural maintenance of chromosomes 3 (SMC3), is located in the basement membrane. The molecule has a complex structure that differs markedly from that of other proteoglycans: the globular or head domains, located at the N- and C-terminuses, are connected via a rod-shaped coiled-coil structure, which is interrupted in the middle by a small site [64,65]. Chondroitin chains are located at the junctions of the head structures with the rod [64]. The SMC3 protein is known to be a part of the cohesin complex necessary for chromosome cohesion and coordinated segregation of sister chromatids [66,67]. All the studied echinoderm species have the bamacan gene. There is a lack of information about the bamacan functions in echinoderms.

#### 2.2.5. Perlecan

Perlecan, or heparan sulfate proteoglycan 2 (HSPG2), is one of the most important components of basement membranes. It is found in a variety of tissues such as cartilage, adipose, nervous, bone, etc. [68]. In most cases, perlecan is a hybrid heparan sulfate/chondroitin sulfate proteoglycan. Its molecule has a modular structure and includes five domains, each of which is capable of binding to many ligands and other molecules. This structure imparts a large number of different functions to HSPG2. For example, domain I binds to growth factors, which contributes to cell proliferation, differentiation, and tissue development; domain IV is necessary for adhesion and scaffolding; and the C-terminal domain V provides cell–ECM interactions and is involved in angiogenesis and regeneration [69]. The *perlecan* gene was found in all the echinoderm species that we studied. It encodes a protein having a domain structure similar to vertebrate perlecans. In the holothurian *C. schmeltzii*, the *perlecan* expression is found in fissioning, but not intact, individuals [20], which indicates its involvement in the connective tissue transformation. However, due to the large number of intermolecular interactions, this proteoglycan can also act as a regulator. Perlecan probably has a structural and regulatory effect on the processes occurring in MCT.

### 2.3. Glycoproteins

#### 2.3.1. Laminins

Laminins (LAMs) are an important structural component of basement membranes and areas of the ECM that come into contact with cells [70]. These glycoproteins are found in almost all tissues. They function as heterotrimers consisting of a combination of various α, β, and γ subunits [70]. The human LAM gene family encodes five α, four β, and three γ subunits. The domain organization of all subunits includes an N-terminal site, usually consisting of a laminin N-terminal (LN) domain, followed by a region of repeats of laminin-type epidermal growth factor (EGF)-like (LE) domains [71]. The array of LE repeats is interrupted by one or two globular laminin type IV (L4 and LF) domains [71]. The presence of alpha-helical sites, which bind three chains with association into a coiled coil, is common to all laminins [71]. In α subunits, unlike β and γ ones, the coiled-coil region at the C-terminus is followed by five laminin G-like (LG) domains [34]. Laminin molecules are capable of self-assembling and, together with collagen type IV, matrix cell receptors and other components, form network structures of basement membranes. LN and LG1-3 domains are responsible for self-assembly: by means of LN, heterotrimers of laminins bind to each other, with C-terminal LG1-3 necessary for their attachment to the cell surface via integrins [71]. Also, the complex domain system of laminins provides interaction with glycoproteins such as agrin, nidogen, and dystroglycan [72,73]. Due to the wide range of intermolecular interactions and the influence on the ECM structure, laminins are involved in a variety of processes such as cell adhesion, migration, and differentiation [70].

The total number of echinoderm LAMs is from five to eight genes, with subunits of all three types occurring among their products. Among the echinoderm classes studied, the highest number of LAM genes were found in holothurians (8) and crinoids (7), while five LAM genes were found in echinoids and asteroids. The laminin expression is positively regulated during fission in the holothurian *C. schmeltzii* [20]. Moreover, along with some other ECM components, laminin shows increased expression during the gut regeneration in the holothurians *Holothuria glaberrima* and *A. japonicus* [74,75]. Together, these facts indicate the important role of laminin in connective tissue transformations, including in MCT during various morphogenesis processes.

#### 2.3.2. Nidogens

Nidogens (NID), or entactins, are a family of glycoproteins located in the basal lamina. Vertebrates have two genes, NID1 and NID2, encoding the proteins. In mammals, serious disorders in the basement membrane structure are observed in case of the absence of both nidogens, which leads to prenatal death in the late stages of lung and heart development [76]. However, with one of the genes working, the structure of the basement membrane is not disturbed, which indicates overlapping functions of their products [76]. Nidogens interact with laminins, perlecan, fibulins, and collagens I and IV. Together, these proteins constitute the basis of the supramolecular structure of basement membranes [77,78]. The relationship of NID1 with the processes of stem cell expansion and differentiation has also been shown [79].

The nidogen molecule consists of three globular domains: G1, G2, and G3. G1 and G2 are separated by a link region, and G2 and G3 by the rod domain. The rod domain contains EGF-like sequences and thyroglobulin-like module (TY) motifs. Also, in the globular domains G2 in NID1 and NID2 and in G3 in NID1, there are EGF repeats that can influence the processes of cell development, adhesion, and protein–protein interactions [80].

**Figure 11:**
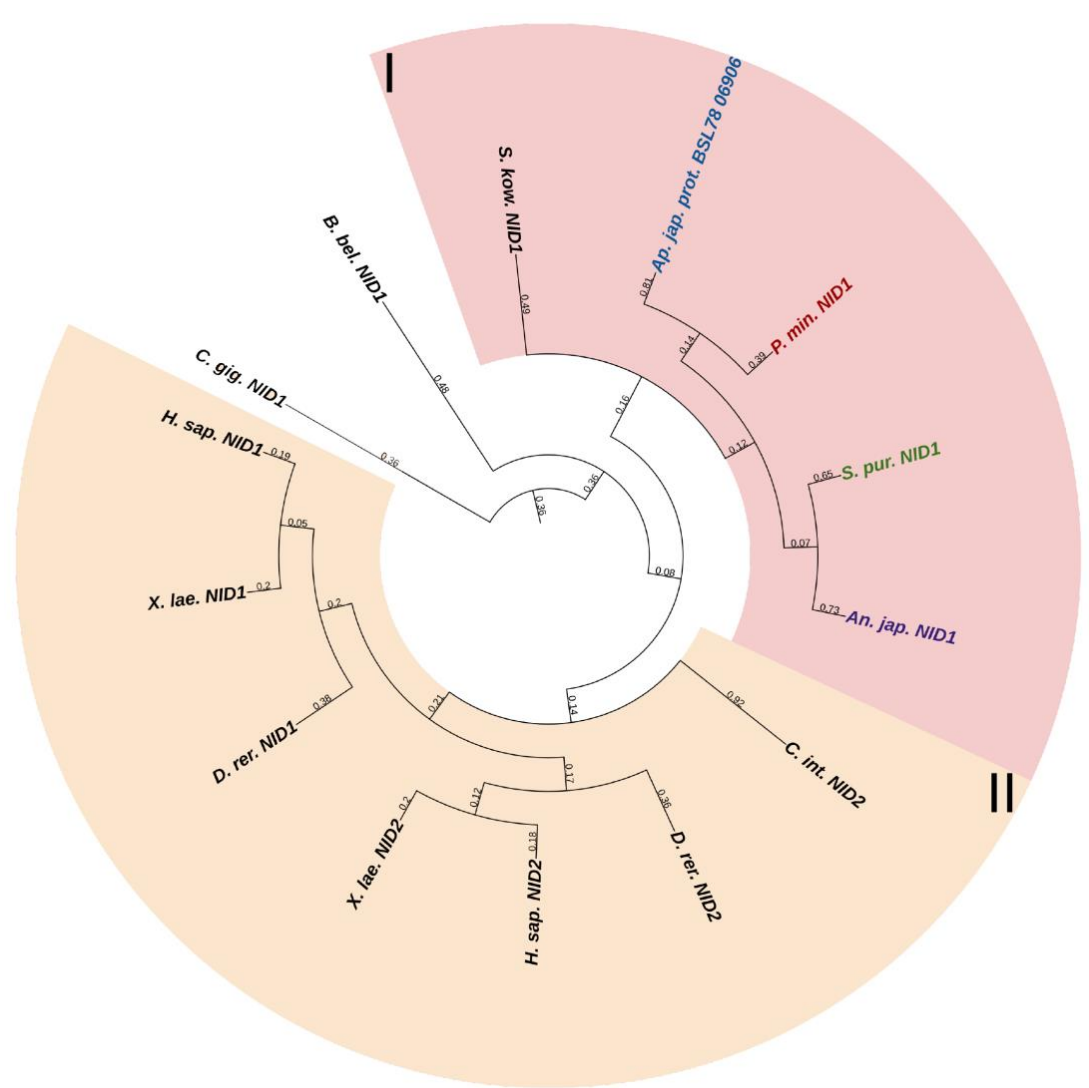
Phylogenetic tree showing the relationships of Nidogens of chordates, hemichordates, and echinoderms. Crinoids (*Anneissia japonica*)—purple color; asteroids (*Patiria miniata*)—red color; echinoids (*Strongylocentrotus purpuratus*)—green color; holothurians (*Apostichopus japonicus*)—blue color; hemichordates (*Saccoglossus kowalevskii*) and chordates (*Ciona intestinalis*, *Branchiostoma belcheri* and *Homo sapiens*)—black color. Groups of proteins (I, II) are marked with colored areas.

Echinoderms have one *NID* gene. The structure of its products is very similar to that of vertebrate nidogens. The difference is the lack of echinoderm TY motifs in the structure of NIDs. In a phylogenetic tree, the echinoderm and vertebrate nidogens form separate groups, which indicates the divergence of these genes as early as at the level of the common ancestor of all deuterostomes (Figure 11, Supplementary File S9). Accordingly, any orthology between them is hardly to be assumed. Nevertheless, judging by the presence of the EGF motif at the end of the G3 region in nidogen sequences of most echinoderms, these are more similar to vertebrate NID1. Taking into account the similarity of the domain organizations, one can assume that nidogens in echinoderms are also one of the most important proteins of basement membranes. In *C. schmeltzii*, nidogen expression is observed in asexual reproduction, while in intact animals, it is absent, which indicates its important role in changes in the connective tissue state [20].

#### 2.3.3. Fibulins

**Figure 12:**
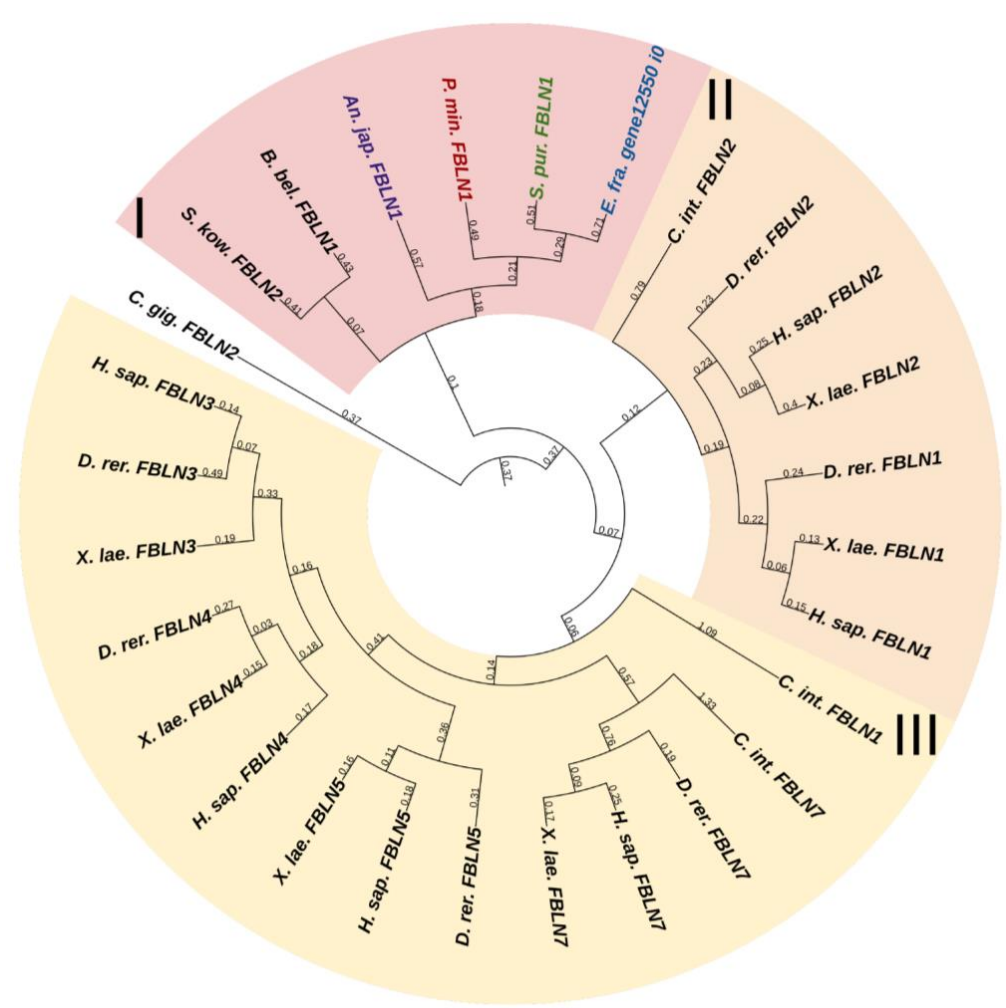
Phylogenetic tree showing the relationships of Fibulins 1, 2, 3, 4, 5, and 7 of chordates, hemichordates, and echinoderms. Crinoids (*Anneissia japonica*)—purple color; asteroids (*Patiria miniata*)—red color; echinoids (*Strongylocentrotus purpuratus*)—green color; holothurians (*Eupentacta fraudatrix*)—blue color; hemichordates (*Saccoglossus kowalevskii*) and chordates (*Ciona intestinalis*, *Branchiostoma belcheri* and *Homo sapiens*)—black color. Groups of proteins (I–III) are marked with colored areas.

**Figure 13:**
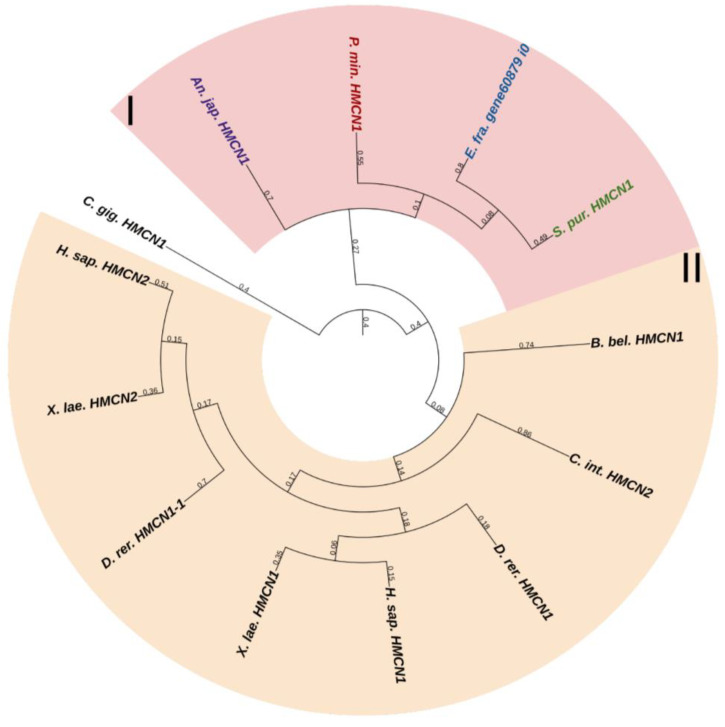
Phylogenetic tree showing the relationships of Fibulins 6 and 8 (Hemicentins) of chordates, hemichordates, and echinoderms. Crinoids (*Anneissia japonica*)—purple color; asteroids (*Patiria miniata*)—red color; echinoids (*Strongylocentrotus purpuratus*)—green color; holothurians (*Eupentacta fraudatrix*)—blue color; hemichordates (*Saccoglossus kowalevskii*) and chordates (*Ciona intestinalis*, *Branchiostoma belcheri* and *Homo sapiens*)—black color. Groups of proteins (I, II) are marked with colored areas.

Fibulins (FBLNs) are a family of basement membrane glycoproteins that have an early evolutionary origin [81]. These are recognized in a variety of taxa, from Cnidaria to Chordata [1,82]. Vertebrates have six to seven FBLN genes. Fibulins, due to their ability to bind to ECM molecules such as fibrillin, are involved in the maintenance of connective tissue supramolecular complexes, e.g., microfibrillar networks [83,84]. On the basis of domain structure and length, two subfamilies of FBLNs can be distinguished: the first comprises FBLN 1,2,3,4,5,7; the second, FBLN 6 and 8, also known as hemicentins (HMCN) 1 and 2. One gene from each subfamily was found in echinoderms (Figure 12 and Figure 13, Supplementary Files S10 and S11). On the phylogenetic tree, the FBLNs of echinoderms and vertebrates form separate groups, which indicates the divergence of these genes at the level of the common ancestor of all deuterostomes.

The echinoderm FBLN, like that of vertebrates, consists of an N-terminal signal peptide, domains of Anaphylotoxin (AT), and EGF repeats. The major function of vertebrate FBLNs is to be involved in cell signaling by interacting with ECM components such as fibronectin, laminin-1, and versican, as well as in tissue renewal through interaction with a disintegrin and metalloproteinase with thrombospondin motifs protease 1 (ADAMTS-1) [83]. During fission in the holothurian *C. schmeltzii*, fibulin-1 shows a positive regulation [20]. Fibulins in echinoderms can be part of the microfibrillar networks of MCT, as well as regulate morphogenetic processes that are accompanied by its change.

The second gene encodes a protein similar to vertebrate hemicentrins. The hemicentrin molecule of both echinoderms and vertebrates consists of an N-terminal signal peptide, a vWA domain, Immunoglobulin C-2 (IGc2) repeats, thrombospondin type 1 repeats, a G2F domain, followed by EGF repeats, and a C-terminal Fibulin-type module. HMCNs are involved in migration processes and provide cell–cell and cell–matrix contacts [85]. Their function in echinoderms remains unknown.

#### 2.3.4. Agrin

Agrin in mammals is an important component of the synaptic basal lamina at the neuromuscular junctions [86,87]. It, as with many other ECM proteins, has a modular structure: a signal peptide and an NtA domain responsible for attachment to the basal lamina via linkage with laminins are located at the N-terminus [88]. Then follows a central region mainly formed by follistatin-like domains that inhibit protease activity [89] and also including other structures: factor I membrane attack complex (FIMAC) and Laminin-type EGF. Behind the central site, there are SEA domains interrupted by EGF sequences and three laminin G-like domains (G1–G3). G1 and G2 domains bind dystroglycan [90], while G3 and, presumably, SEA are required for aggregation and clustering of acetylcholine receptors at the neuromuscular junctions [91,92]. In vertebrates, agrin is involved in the formation, maintenance, and regeneration of neuromuscular junctions [93,94,95].

Echinoderm agrins generally manifest a structure similar to that of mammalian agrins. However, in echinoderms, this protein has a number of specific features. It lacks SEA domains and has structures containing cysteine repeats. Also, the NtA domain has not been found in agrins of most echinoderms. The exceptions are the holothurians *E. fraudatrix* and *H. leucospilota* (agrin of *A. japonicus* lacks part of the sequence at the N-terminus) and the sea star *A. planci*. At the N-terminuses of agrins in the sea stars *A. rubens* and *P. miniata*, there is a different structure, the transmembrane transport protein Major Facilitator Superfamily (MFS), while agrins of sea urchins immediately begin with follistatin-like domains. In the holothurian *C. schmeltzii*, *agrin* is active in the body wall of intact animals, but its expression is not detected in fissioning individuals [20]. It is possible that agrin is involved in the formation and maintenance of juxtaligamental cells, which are the effectors directly responsible for changes in MCT tensility [13].

#### 2.3.5. Dystroglycan

Dystroglycan is a receptor for cell adhesion of ECM proteins. It presents in a variety of tissues, both during embryonic development and in the postnatal period [96,97]. Echinoderms, like vertebrates, have one *dystroglycan* gene, DAG1. It encodes a precursor, which, after translation, is cut into two subunits subsequently forming a non-covalent bond with each other [98]. The first subunit, extracellular αDG, consists of a glycosylated mucin-like domain flanked by two globular domains. The second, βDG, has transmembrane and cytoplasmic domains [98]. The mucin-like domain of αDG interacts with laminins, agrin, perlecan, and other ECM proteins in muscle and nervous tissues [98]. Dystroglycan is necessary to maintain the structural integrity of basement membranes and the central nervous system development [99,100,101]. DAG1 is expressed by neuroepithelial cells and regulates the migration of glial cells [101]. Dystroglycan is also involved in dendritogenesis and axon guidance, and, along with agrin, it is involved in the formation of neuromuscular junctions [101]. DAG1 in echinoderms probably performs similar functions. It can, together with agrin, be involved in the formation of neural connections in MCT. This may indirectly be indicated by the fact that in *C. schmeltzii* the activity of dystroglycan in the body wall, like that of agrin, disappears during fissioning [20].

#### 2.3.6. Thrombospondins

Thrombospondins (THBSs) are a family of multidomain, multifunctional, calcium-binding glycoproteins that interact with cell surfaces, other ECM components, growth factors, cytokines, and proteases [1]. Vertebrates have five genes of THBSs. THBSs can be divided into two groups depending on the quaternary structures that they form. Group A contains proteins THBS1 and THBS2, which form trimers, and group B contains THBS3, THBS4, and THBS5, which form pentamers [102]. The THBS-A molecule includes many domains: a N-terminal domain (THBS-N), a coiled-coil domain, a vWC domain, three properdin-like repeats or thrombospondin repeats (TSRs), a signature domain comprising three EGF repeats, a calcium-binding wire, and a lectin-like C-terminal globe [102]. THBS-B has a similar structure, except for the absence of vWC and TSRs and the presence of an additional EGF repeat [102]. Also, THBS-5 lacks THBS-N [102].

**Figure 14:**
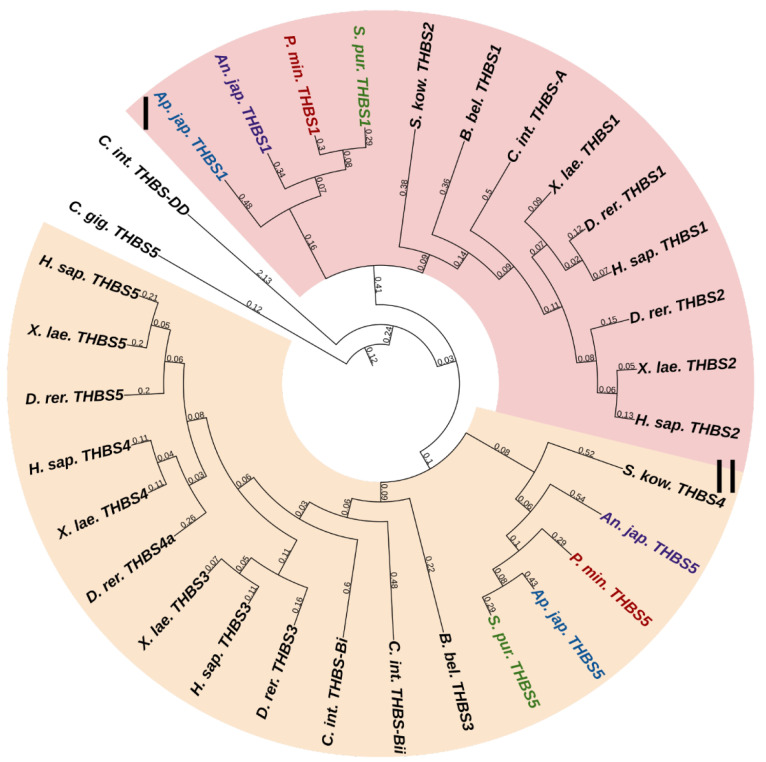
Phylogenetic trees showing the relationships of thrombospondins of chordates, hemichordates, and echinoderms. Crinoids (*Anneissia japonica*)—purple color; asteroids (*Patiria miniata*)—red color; echinoids (*Strongylocentrotus purpuratus*)—green color; holothurians (*Apostichopus japonicus*)—blue color; hemichordates (*Saccoglossus kowalevskii*) and chordates (*Ciona intestinalis*, *Branchiostoma belcheri* and *Homo sapiens*)—black color. Groups of proteins (I, II) are marked with colored areas.

Two THBS genes have been found in all echinoderms (Figure 14, Supplementary File S12). The first encodes a protein that shows similarities to THBS 1 and 2 in vertebrates, and therefore, it can be designated as THBS1/2. In mammals, these THBSs are involved in ECM assembly, platelet aggregation, inflammatory response, and regulation of angiogenesis during regeneration and tumor growth [103]. Moreover, they perform a structural function in ECM, acting as molecular bridges between its components. THBS1 and 2 are capable of binding to integrin and fibrillar collagens [104,105,106].

The second echinoderm gene is referred to as *THBS5* or *Cartilage oligomeric matrix protein* (*COMP*) in the NCBI database. The protein encoded by it has a domain structure corresponding to the THBS-B group. In this regard, this gene can be referred to as *THBS3/4/5*. Vertebrate THBS5 can form molecular bridges between ECM proteins [103], in particular, by binding to FACITs [107,108]. In echinoderms, thrombospondins probably perform a similar function. By interacting with collagen fibrils, they can be involved in the mechanisms changing the mechanical properties of MCT.

#### 2.3.7. Fibrillins

Fibrillins (FBNs) are among the most ancient proteins capable of forming fibrils in ECM [1]. In vertebrates, these are widely known as components critically important for composing fibers that form a scaffold for elastin [109]. Vertebrate FBNs are structurally similar to each other and consist of about 30 EGF modules, which provide, first, stabilization of the protein structure, second, its protection from proteolysis, and third, interaction with the ECM components, e.g., with fibulin [110]. Among EGF modules, there are about eight transforming growth factor beta (TGF)-β binding protein (TB)-like modules, mediating reactions with integrins [110], and one to three vWA domains. Fibrillin 1 unique N-terminal domain (FUN), which, interacting with the C-terminal site of another FBN molecule, provides assembly of microfibrils, is located at the N-terminus [111]. The C-terminal domain is also capable of binding ECM proteins [110].

**Figure 15:**
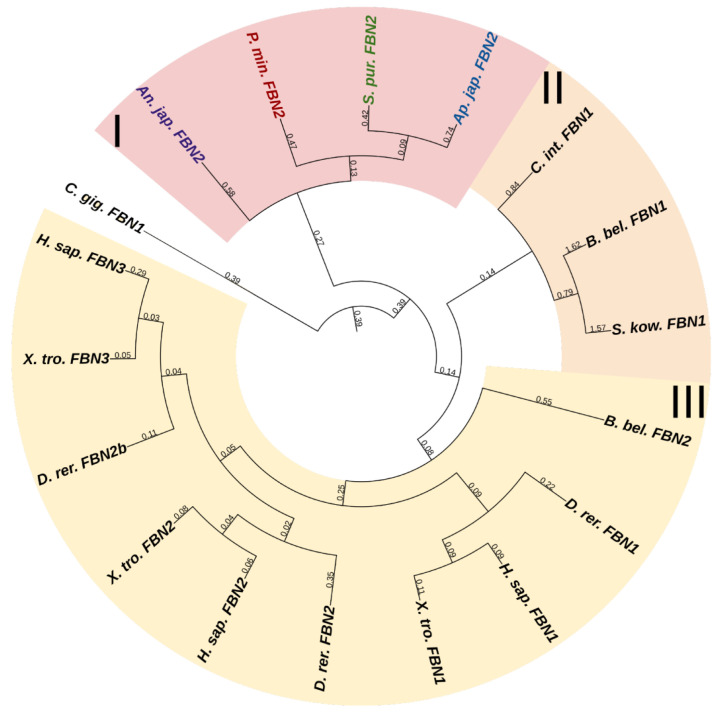
Phylogenetic trees showing the relationships of fibrillins of chordates, hemichordates, and echinoderms. Crinoids (*Anneissia japonica*)—purple color; asteroids (*Patiria miniata*)—red color; echinoids (*Strongylocentrotus purpuratus*)—green color; holothurians (*Apostichopus japonicus*)—blue color; hemichordates (*Saccoglossus kowalevskii*) and chordates (*Ciona intestinalis*, *Branchiostoma belcheri* and *Homo sapiens*)—black color. Groups of proteins (I–III) are marked with colored areas.

Vertebrates have three genes of this family; echinoderms, according to the NCBI database, have from two to three. One of them, more frequently designated as FBN2 and present in all the echinoderms species that we studied, has a domain organization similar to that of vertebrate fibrillins and includes all of the above structures in comparable amounts. The rest of the echinoderm sequences found, though confidently identified by BLAST as fibrillins, have a slightly different structure. First, these do not contain the FUN domain, and second, these sequences differ substantially from each other by the absence of TB modules or vWA domains or, vice versa, by the presence of other structures. In the transcriptome of the holothurian *E. fraudatrix*, we found about six structurally different sequences that are identified as fibrillins by BLAST. This is probably explained by the fact that the vast majority of domains in fibrillins are not unique to them and are present in other proteins, which hampers their accurate identification. For example, a very similar arrangement of EGF and TB modules is found in the latent-transforming growth factor beta-binding protein 1 that is absent in echinoderms. Thus, we assume that echinoderms possess only a single *fibrillin* gene (Figure 15, Supplementary File S13). The rest of the FBN-like proteins probably represent a separate group of connective tissue proteins that has formed within the phylum Echinodermata.

Besides being involved in elastogenesis, FBNs perform other functions that are of greater interest within the scope of this review, because echinoderms lack elastins. Thus, they are involved in the assembly of microfibrillar networks and basement membranes; they can influence growth factors and are, thereby, indirectly involved in the regulation of cell functions and stem cell niches, as well as provide cell–matrix communication by interacting with integrins [85]. Fibrillin molecules are present in MCT of the holothurian body wall. Studies using electron microscopy have shown that fibrillins in MCT form a microfibrillar network around collagen fibers [112]. Stretching of MCT is accompanied by the sliding of collagen fibers relative to each other, and the microfibrillar network provides them with the correct orientation during movement. It has also been shown that the microfibrillar network isolated from the body wall of the holothurian *Cucumaria frondosa* (Gunnerus, 1767) behaves as a highly extensible elastomer [112]. Thus, fibrillin, as a component of microfibrillar networks, imparts on MCT the ability to stretch and the strength, and is also involved in the correct orientation of collagen fibers in MCT.

#### 2.3.8. SPARC-Related Modular Calcium Binding Protein 1

SPARC-Related Modular Calcium Binding protein 1 (SMOC) is a family of proteins located in basement membranes and ECM [113]. Vertebrates have two genes coding proteins of this family. Structurally, SMOC1 and SMOC2 are similar and have a follistatin-like domain, two thyroglobulin-like domains, a unique domain, and an extracellular calcium-binding domain [113]. SMOCs are involved in a variety of processes such as migration, proliferation, cell adhesion, tissue fibrosis, angiogenesis, calcification, and tumor development [113]. Only one gene encoding SMOC with all the above-listed domains has been identified in echinoderms. Judging by the similarity of structures of SMOC in echinoderms, it probably has a similar set of functions and may be involved in connective tissue remodeling.

### 2.4. Polysaccharides

Connective tissue of animals includes diverse GAGs such as hyaluronic acid, chondroitin sulfates, and heparan sulfates. Their synthesis requires various enzymes. Hyaluronic acid is synthesized by the enzymes hyaluronan synthases. Mammals have three genes *hyaluronan synthase*, while invertebrates lack them [114]. We have also not found *hyaluronan synthases* in echinoderms. This is a noteworthy finding because these animals have hyaluronidase, an enzyme that, in mammals, breaks down hyaluronic acid into monosaccharides. However, among invertebrates, hyaluronic acid has been found only in mollusks [114], which suggests that hyaluronidase in echinoderms has a different substrate specificity.

The chondroitin sulfate biosynthesis involves some enzymes of the Beta 4-glycosyltransferases family, e.g., chondroitin sulfate synthases (Chsy) [115]. In echinoderms, as well as in vertebrates, two *Chsy* genes have been found. The synthesis of heparan sulfates in vertebrates is provided by the enzymes EXTL1, EXTL2, and EXTL3, which constitute the Exostosin glycosyltransferase family (EXT) [116]. Echinoderms also have three *EXT* genes.

Chondroitin sulfates and heparan sulfates are part of proteoglycans, are required for cell hydration, structural scaffolding, and also play a key role in cell signaling [117]. It has been shown that in MCT structures of crinoids, echinoids, and holothuroids, highly sulfated chondroitin sulfates are located along collagen fibrils [118,119,120,121]. In this regard, the enzymes that synthesize and degrade GAGs can play an important role in the modification of MCT in echinoderms.

## 3. Proteins Modifying ECM

### 3.1. Collagen Formation

The synthesis of ECM and change in its properties depend, first, on the enzymes responsible for the assembly of various types of fibrils that constitute the basis of connective tissue. Transglutaminase-2 [122] and lysyl oxidase (Lox) are involved in the formation of collagen fibrils [123,124]. An analysis of the NCBI databases has shown that echinoderms possess one *Lox* and some *transglutaminase* genes.

#### 3.1.1. Lysyl Oxidase

**Figure 16:**
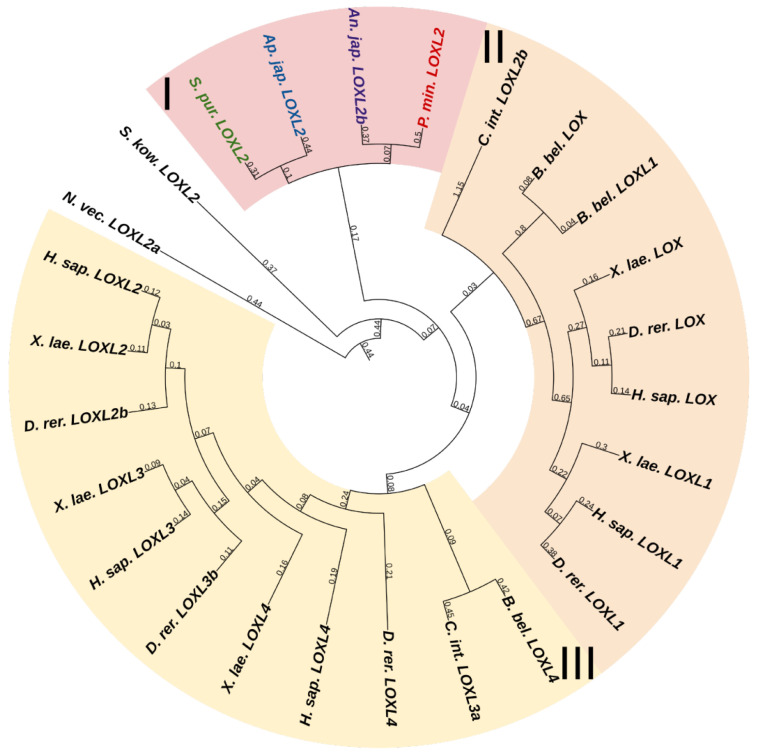
Phylogenetic trees showing the relationships of Lysyl oxidase of chordates, hemichordates, and echinoderms. Crinoids (*Anneissia japonica*)—purple color; asteroids (*Patiria miniata*)—red color; echinoids (*Strongylocentrotus purpuratus*)—green color; holothurians (*Apostichopus japonicus*)—blue color; hemichordates (*Saccoglossus kowalevskii*) and chordates (*Ciona intestinalis*, *Branchiostoma belcheri* and *Homo sapiens*)—black color. Groups of proteins (I–III) are marked with colored areas.

Lysyl oxidases (LOXs) are a family of copper-dependent amino oxidases capable of ECM remodeling by forming various inter- and intra-chain cross-links in collagens and elastins [125]. LOXs can oxidize lysine and hydroxylysine residues to reactive aldehyde species that eventually form associations with other oxidized groups or intact lysines [125]. Vertebrates have five *LOXs* genes, while echinoderms have only one (Figure 16, Supplementary File S14). Apparently, *LOXs* of deuterostomes diverged from a single ancestral gene during the divergence of Ambulacraria and Chordata. The function of LOX in echinoderms most likely does not differ from that in vertebrates and consists of establishing cross-links between collagens.

#### 3.1.2. Transglutaminases

**Figure 17:**
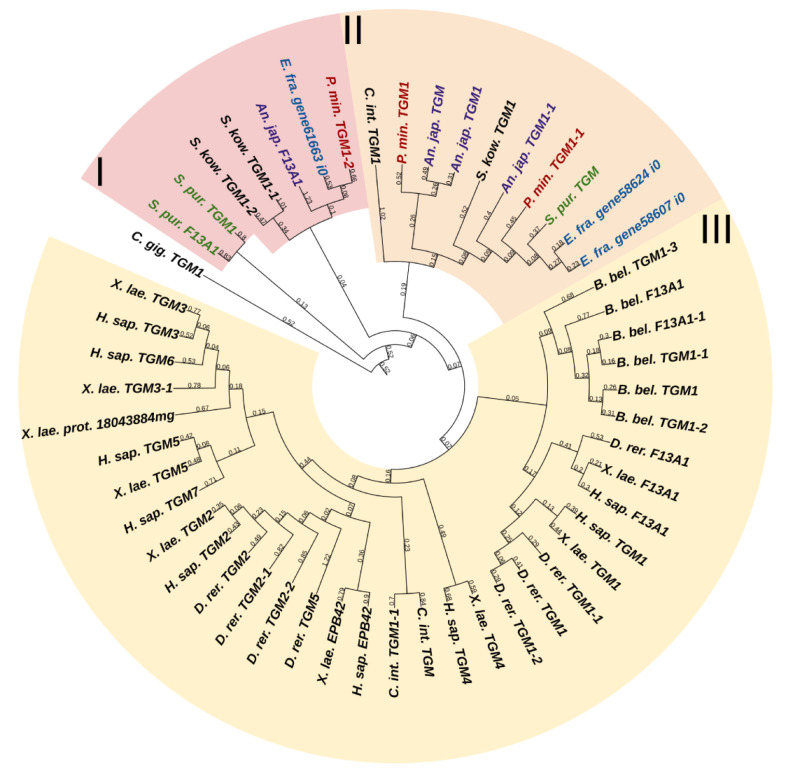
Phylogenetic trees showing the relationships of transglutaminases of chordates, hemichordates, and echinoderms. Crinoids (*Anneissia japonica*)—purple color; asteroids (*Patiria miniata*)—red color; echinoids (*Strongylocentrotus purpuratus*)—green color; holothurians (*Eupentacta fraudatrix*)—blue color; hemichordates (*Saccoglossus kowalevskii*) and chordates (*Ciona intestinalis*, *Branchiostoma belcheri* and *Homo sapiens*)—black color. Groups of proteins (I–III) are marked with colored areas.

Transglutaminases (TGM) are a family of Ca^2+^-dependent enzymes that covalently bind amino groups of one protein to the γ-carboxamide groups of glutamines of another [126]. Vertebrates have eight to nine *TGM* genes that perform many functions in various tissues such as apoptosis, adhesion, ECM stabilization, signal transmission, coagulation of germ and blood cells, and formation of bone tissue and cell membrane of keratinocytes [127]. One protein from this family, EPB42, does not have catalytic activity but is, nevertheless, involved in signaling, structural scaffolding, and adhesive functions [127]. The domain structures of all vertebrate TGMs are similar and include the N-terminal, catalytic middle domains, and one or two domains located at the C-terminal site. Transglutaminases of echinoderms have a structure identical to those of vertebrates. Three *TGM* genes have been found in all classes, except for crinoids, which have four genes (Figure 17, Supplementary File S15). In a phylogenetic tree, all the echinoderm TGMs are grouped separately from vertebrate TGMs, which indicates an earlier divergence from the ancestral gene and the lack of correspondence between them. It has been shown that transglutaminases in sea urchins are involved in embryonic development and affect cell proliferation [128]. Also, as evidenced by experiments with their inhibitors, transglutaminases probably cause stiffening of MCT [129].

### 3.2. Proteases

Animals possess a wide variety of proteases capable of degrading ECM proteins. These are serine, cysteine, aspartyl, and metal peptidases. The transformation of connective tissue during fission in echinoderms has been shown to be accompanied by variation in the expression of genes of numerous proteases and their inhibitors such as matrix metalloproteinases (MMPs), ADAMTSs, a tissue inhibitor of metalloproteinases (TIMPs), and Cathepsin D [20]. This emphasizes the importance of these proteins in morphogenetic processes accompanied by rearrangements in connective tissue.

#### 3.2.1. Serine Proteases

The family of serine proteinases comprises a large number of proteolytic enzymes involved in many biological processes [130]. It is known that many serine proteases of various types can degrade connective tissue proteins. These include plasmin, cathepsin G, fibroblast activation protein α, kallikrein 12 (KLK12), neurotrypsin, furin, matriptase (ST14), hepsin, neutrophil elastase, activated protein C, KLK 4 and 14, etc. [131,132,133]. They can lyse ECM components directly or indirectly, by activating other proteinases such as MMP [71,72]. Of all the above proteins, only furin is reliably identified in echinoderms.

The domain organization of furins does not fundamentally differ between vertebrates and echinoderms. The N-terminal signal peptide and the subsequent propeptide are involved in successive posttranslational modifications such as proteolytic cleavage, glycosylation, and folding [134]. A catalytic, P domain stabilizing it, and a cysteine-rich domain, are located next [134]. These are followed by a cytoplasmic domain responsible for protein localization and a transmembrane domain. Removing the propeptide, furin activates extracellular proteases MMPs and a disintegrin and metalloproteinases (ADAMs) by cleaving the molecule in the region of furin-activated motif (R-X-R/K-R) [135]. In addition, it is involved in the maturation of some of ECM components: collagen type V, XIII, XXV [26,136,137] (see above), integrins, and various growth and differentiation factors [134,138].

Besides furin, proteases referred to in NCBI as serine proteases have been identified in echinoderms. Eight serine proteinases have been found in the holothurian *C. schmeltzii*, of which four are expressed only in fissioning individuals [20]. A study on the holothurian *A. japonicus* has shown that serine proteases are capable of effectively degrading collagen [139,140]. Thus, an assumption can be made that in echinoderms, serine proteinases are involved in the processes of connective tissue remodeling.

The use of BLAST on echinoderms has shown proteases Hepsin and KLK4, 12 and 14 to best match with the sequences designated as trypsins. However, a search for these “trypsins” among mammals provides ambiguous matches, which hampers identification of these proteins and assumption on their functions. The situation is similar to that with other serine proteases, e.g., plasmin. The systematics of echinoderm serine proteases requires dedicated studies, which may identify new candidates to the role of ECM remodeling among proteins of this family.

#### 3.2.2. Cysteine Proteases

Cysteine proteases are a group of enzymes that play a major role in a variety of biological processes including digestion, apoptosis, and protein processing. These enzymes are characterized by the nucleophilic cysteine residue in the active center that catalyzes the hydrolysis of peptide bonds [141]. One of the subgroups of cysteine proteases is cathepsins (CTS). As a rule, these have intracellular localization, but some of them (cathepsins B, L, K and S) can be secreted into the intercellular space and degrade ECM proteins [142,143]. For example, STSK is secreted by osteoclasts and is involved in bone remodeling. Among ECM proteins, the substrates for the listed proteinases are collagens I, II, and IV, aggrecan, perlecan, nidogen, and laminin [142].

Cysteine cathepsins have a similar structure: a signal peptide is located at the N-terminus, followed by the propeptide inhibitor I29 required for post-translational modifications and activation, and then by the peptidase domain C1 containing a catalytic site [144]. Of cathepsins capable of degrading extracellular components, CTSB and CTSL were identified in echinoderms. In *A*. *japonicus*, the cathepsin L-like protein is found in the outer layer of dermis [134]. It is assumed to be involved in the autolysis of the body-wall connective tissue in holothurians. In *C*. *schmeltzii*, *cathepsin L* is expressed during asexual reproduction [20]. CTSB may be involved in regenerative processes in echinoderms, as it has been detected in spines of the sea urchin *Echinometra lucunter* capable of regeneration [145]. Thus, cathepsins should be taken into account when analyzing the mechanisms changing the MCT properties, since these proteases can be involved in ECM remodeling in echinoderms.

#### 3.2.3. Aspartyl Protease

Aspartyl proteases represent a group of peptidases that cleave protein substrates using two aspartic acid residues located in their catalytic center [146]. One of proteases of this type is cathepsin D. It is a lysosomal enzyme, but it can also be localized in the extracellular space [147], where it can cleave aggrecan molecules [148]. The CTSD structures in echinoderms and vertebrates are similar and do not fundamentally differ from the structural organization of cysteine cathepsins. There is evidence of the involvement of CTSD in morphogenetic processes in holothurians. In *A. japonicus*, it is involved in autolysis of body wall, muscles, and gut [149]. In *C. schmeltzii*, CTSD begins to be expressed in the constriction area of fissioning individuals [20]. Thus, CTSD is likely to be involved in processes of MCT transformation.

#### 3.2.4. Matrix Metalloproteinases

Among the enzymes involved in the ECM remodeling, proteases of the metzincin superfamily are of particular interest [150]. This group includes most of the well-known metalloendoproteinases: matrix metalloproteinases (MMPs), a disintegrin and metalloproteinases (ADAMs), a disintegrin and metalloproteinase with thrombospondin motifs (ADAMTSs), pappalysins (pregnancy-associated plasma proteins), serralysins (bacterial enzymes), leishmanolysins (protozoan proteinases), and astacins [150,151]. All of them contain zinc in the active center. Many of these proteases are involved in ECM degradation, but the most significant group of enzymes involved in connective tissue remodeling is MMPs, also referred to as matrixins [152]. Depending on their specialization, MMPs can either degrade extracellular matrix components or perform site-specific proteolysis by activating or inactivating various proteins [153,154]. The number of *MMPs* varies between different echinoderm species. In the *A. japonica* genome, a total of 22 *MMP* genes have been identified; in *P. miniata*, 20 *MMPs*; in *S. purpuratus*, 21 *MMPs*; and in *A. japonicus*, 18 [155]. These are comparable to the number of *MMPs* genes in vertebrates (25–33). The structure and functions of echinoderm MMPs are described in detail in the review by Dolmatov et al. [155]. MMPs are assumed to play an important role in the mechanisms changing the mechanical properties of MCT [13,156]. Galardin (the synthetic MMP inhibitor) stiffens ligaments in sea urchins [156]. Furthermore, MMPs are involved in dermal liquefaction in holothurians [157,158].

#### 3.2.5. ADAMs and ADAMTSs

Humans have 21 *ADAMs* and 19 *ADAMTSs* [159]. ADAMs and ADAMTs are anchored on the cell membrane. They differ from MMPs by the absence of hemopexin-like repeats and the presence of EGF-like and disintegrin domains. ADAMTSs also have thrombospondin repeats [159]. The number of *ADAMTS* and *ADAM* genes in echinoderms differs between members of different classes. The greatest number of *ADAMTS* genes (14) have been found in the crinoid *A. japonica*, the smallest number being found in the holothurian *A. japonicus* and the sea urchin *S. purpuratus*, with 11 in each. The sea star *P. miniata* has 12 *ADAMTS* genes. Five genes encoding ADAM have been identified in each of the echinoderm species studied.

Unlike ADAMs, ADAMTSs are mostly specialized in degradation of ECM components and are, thus, actively involved in the processes of cell migration, proliferation, and differentiation [160]. They cut N-propeptides of collagens I and II, thereby being involved in the assembly of collagen fibrils [161]. ADAMTSs also cut off the prodomains in some of proteoglycans (aggrecan, versican, brevican, and neurocan) and glycoprotein COMP [160]. Furthermore, fibulins, TGFbRIII, LOX, perlecan, and THBS-1 may be potential substrates for ADAMTSs [162]. ADAMTSs in echinoderms may be involved in the processes of degradation of ECM components in MCT. Seven transcripts of *ADAMTs* have been identified in *C. schmeltzii* [20]. ADAMTS7 and ADAMTS9 are positively regulated in fissioning individuals, while ADAMTS13 and ADAMTS14, vice versa, are negatively regulated. 

## 4. Regulation of Protease Activity

Each class of proteases has its own specific inhibitors [163]. However, examples of proteins are known that can inhibit a serine and metalloprotease [164,165], cysteine and aspartic protease [166], or a serine protease and amylase [163,167,168].

### 4.1. Serine Protease Inhibitors

Serine proteinase inhibitors, or SERPINs, are a superfamily of proteins, of which most are capable of inhibiting proteinases [169]. SERPINs are divided into nine clades designated with letters from A to I; in humans, it includes a total of 36 genes. Many SERPIN proteins are involved in the processes of connective tissue transformation [169]. Only SERPINB has been found in echinoderms. SERPINB1, or Leukocyte Elastase Inhibitor (LEI), is an inhibitor of trypsin [170] and elastase [171], which is absent from echinoderms. In addition to serine proteases, this protein can inhibit aspartyl and cysteine proteases such as cathepsin D and cathepsin L [169,171]. Also, several vertebrate cathepsins involved in ECM remodeling, including cathepsin L, are regulated by the SERPINB3 protein [169]. There is evidence of the ability of LEI to mediate negative regulation of MMP2 in vertebrates [172]. Another vertebrate protease from this clade, SERPINB8, inhibits the action of furin required for the MMP activation [169]. Thus, the role of SERPINs in connective tissue of echinoderms may be related to the work of serine proteinases, cathepsin L, and furin.

### 4.2. Metalloproteinase Inhibitors

The major MMP inhibitors are *α*-2-macroglobulin, reversion-inducing cysteine-rich protein with Kazal motifs (RECK), and tissue inhibitors of metalloproteinases (TIMPs). Our study showed that echinoderms lack *α*-2-macroglobulin. RECK is an important participant in the process of connective tissue remodeling. Its main function is the inhibition of MMP2, MMP9, and MT1-MMP [173,174]. RECK is a membrane-anchored glycoprotein [175] that has a modular structure, with hydrophobic regions located at the ends of the molecule [176]. The C-terminal site provides binding to the membrane through interaction with glycosylphosphatidylinositol [176]. Closer to the N-terminus, there is a region with five cysteine repeats, which has glycosylation sites required for proper interaction with MMP [176]. It is followed by two central EGF modules, of which one is flanked by three serine-protease inhibitor-like domains SPIs. These modules perform a catalytic function [176]. A gene encoding RECK was found in all echinoderms. It is obviously capable of contributing to MCT transformation by regulating the MMP action.

TIMPs are endogenous inhibitors of a wide spectrum of metalloproteinases from the families MMP, ADAM, and ADAMTS [177]. In this regard, TIMPs play an important role in the regulation of ECM remodeling and turnover. Unlike vertebrates that have only four *TIMP* genes, echinoderms have a large number of genes encoding TIMPs and TIMP-like proteins [155,178]. In some species, the number of such genes can reach 45 [178]. Most of the studied echinoderm TIMPs have a standard structure similar to that of other animals [155,178]. Only one domain is identified in them, the NTR domain, which is characteristic of this protein class. The majority of studied echinoderm TIMPs contain 10–12 conserved cysteine residues, which probably form a tertiary structure of the molecule [20,155,178]. The structure of the echinoderm TIMPs is described in detail in the review by Dolmatov et al. [155].

The functions of TIMPs in echinoderms have not been studied to date. It is obvious that, as in mammals, many of these proteins are MMP inhibitors. Information is only available about tensilins, a specific group of TIMP-like proteins [20,155,179]. Tensilin was first found in the connective tissue of the body wall of the holothurian *C. frondosa* [180]. Later, similar proteins were found in other holothurians [155,179,181]. These proteins are assumed to be responsible for MCT stiffening and are an important component of the mechanism of regulation of MCT properties in echinoderms [49,156,182].

**Figure 18:**
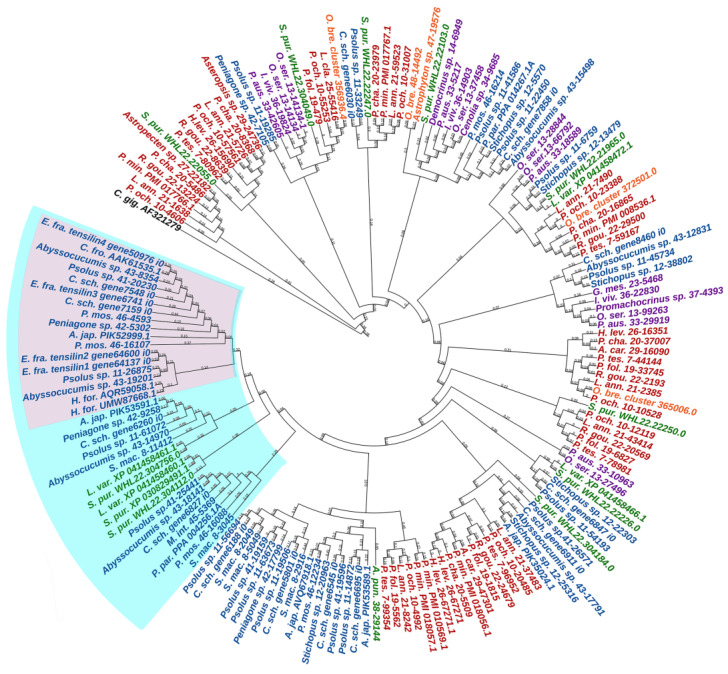
Phylogenetic trees showing the relationships of TIMPs of echinoderms. Crinoids—purple color; asteroids—red color; ophiuroids—orange color; echinoids—green color; holothurians—blue color. A. car.—*Asteropsis carinifera*; A. jap.—*Apostichopus japonicus*; A. pun.—*Arbacia punctulata*; C. fro.—*Cucumaria frondosa*; C. gig.—*Crassostrea gigas*; C. sch.—*Cladolabes schmeltzii*; E. fra.—*Eupentacta fraudatrix*; H. for.—*Holothuria forskali*; H. lev.—*Henricia leviuscula*; G. mes.—*Gephyrocrinus messing*; I. viv.—*Isometra vivipara*; L. ann.—*Labidiaster annulatus*; L. cla.—*Luidia clathrata*; L. var.—*Lytechinus variegatus*; M. int.—*Molpadia intermedia*; O. bre.—*Ophioderma brevispinum*; O. ser.—*Oligometra serripinna*; P. aus.—*Ptilometra australis*; P. cha.—*Psilaster charcoti*; P. fol.—*Peribolaster folliculatus*; P. min.—*Patiria miniata*; P. mos.—*Pannychia moseleyi*; P. och.—*Pisaster ochraceus*; P. par.—*Parastichopus parvimensis*; P. tes.—*Pteraster tesselatus*; R. gou.—*Remaster gourdoni*; S. mac.—*Synapta maculata*; S. pur.—*Strongylocentrotus purpuratus*. Group of tensilins is marked with pink area; group of tensilins and tensilin-like proteins is marked with cyan area.

An analysis of the TIMP phylogeny has shown that tensilins are found only in holothurians [20]. However, Mittal et al. [183] used EchinoDB to find sequences similar to holothurian tensilins in representatives of all classes of echinoderms. To eliminate this contradiction, we analyzed the known sequences of TIMP and TIMP-like proteins of echinoderms, indicated in previously published papers [20,178,181,183]. The phylogenetic tree (Figure 18, Supplementary File S16) confirms our conclusion about the strong divergence of the ancestral *TIMP* gene in the phylogeny of echinoderms [155]. In addition, all holothurian tensilins form a separate group. The closest tensilin homologues are found only in echinoids. Together they form a common cluster including tensilins and tensilin-like proteins. TIMPs and TIMP-like proteins of other echinoderm classes are located outside of this cluster.

**Figure 19:**
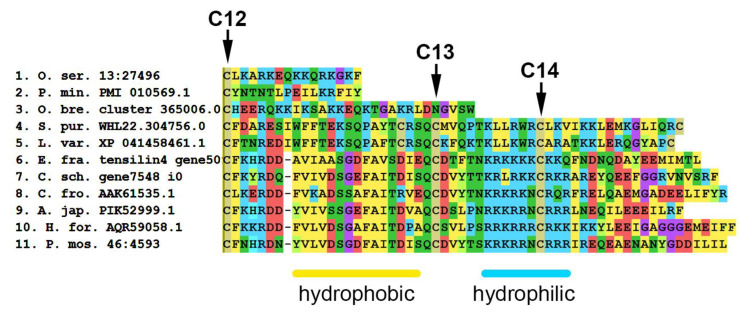
MUSCLE (MEGA) alignment of amino acid sequences of C-terminal part of some tensilins, tensilin-like proteins and TIMPs of echinoderms. C12–C14—conserved cysteine residues. A. jap.—*Apostichopus japonicus*; C. fro.—*Cucumaria frondosa*; C. sch.—*Cladolabes schmeltzii*; E. fra.—*Eupentacta fraudatrix*; H. for.—*Holothuria forskali*; L. var.—*Lytechinus variegatus*; O. bre.—*Ophioderma brevispinum*; O. ser.—*Oligometra serripinna*; P. min.—*Patiria miniata*; P. mos.—*Pannychia moseleyi*; S. pur.—*Strongylocentrotus purpuratus*.

An amino acid sequence analysis shows that tensilins have two distinct features. Most of these proteins have two or three amino acid residues between the first and second cysteines of the N-terminal region of the molecule [155]. In addition, in tensilins, the C-terminal part of the molecule contains two regions —“hydrophobic” and “hydrophilic” (Figure 19, Supplementary File S16). The “hydrophobic” region is located immediately after the 12th cysteine and contains phenylalanine (F), alanine (A), valine (V), leucine (L), and isoleucine (I) residues. This is followed by a “hydrophilic” region in which two more conserved cysteine residues are found. Perhaps they are also involved in the formation of the tertiary structure of the molecule. The fourteenth cysteine is surrounded by several lysine (K) and arginine (R) residues.

Tensilin-like sequences of crinoids, ophiuroids, and asteroids found in EchinoDB are quite short and are aligned only on the N- or C-terminal part of tensilins (see Supplementary File S17). No tensilin-like genes were found in well-annotated genomes of crinoid *A. japonica* and asteroid *P. miniata* [155]. Most likely, these are incomplete transcripts of some other proteins. At the same time, some proteins found in echinoids are structurally similar to holothurian tensilins. These sequences have 2–3 amino acid residues between 1 and 2 cysteines and “hydrophobic” and “hydrophilic” regions at the C-terminus of the molecule. However, in echinoids in the “hydrophilic” region, hydrophobic amino acid residues such as alanine, valine, and leucine are noted among lysine and arginine. How these substitutions affect protein function is unknown.

Despite the supposed importance of tensilins in the mechanisms of changing the properties of MCT, almost nothing is known about the functions of these proteins. Some of them are localized in juxtaligamental cells, a MCT-specific cell type [179,182]. It was shown that the recombinant tensilin of *Holothuria forskali* is able to stiffen dermal pieces and aggregate collagen fibrils [182]. It is assumed that it can form cross-links between collagen fibrils and thereby increase the stiffness of the connective tissue.

Transcripts of four tensilin genes were identified in the transcriptome of the holothurian *E. fraudatrix* [181]. The expression of one of them (*Ef-tensilin3*) increased during the regeneration of the digestive system. Its transcripts were localized in the coelomic and intestinal epithelia of the gut anlage. It is possible that Ef-tensilin3 may be an inhibitor of the Ef-MMP16 proteinase.

The ancestral gene of tensilin, apparently, was repeatedly duplicated, since some of holothurian species have several of its orthologs. The repeated duplication and preservation of orthologs in phylogenesis show that tensilins play an important role in the physiology of holothurians. However, their specific functions in holothurians remain unknown. Apparently, echinoids have orthologs of the tensilin gene. However, similar genes in crinoids, asteroids, and ophiuroids have not been found to date.

## 5. Materials and Methods

For the analysis, we used the genomes of representatives of four echinoderm classes: the crinoid *Anneissia japonica* (Müller, 1841) (PRJNA615663), the sea star *Patiria miniata* (Brandt, 1835) (PRJNA683060), the sea urchin *Strongylocentrotus purpuratus* (Stimpson, 1857) (PRJNA13728), and the holothurian *Apostichopus japonicus* (Selenka, 1867) (PRJNA354676). Sequences of some genes obtained from the *A. japonicus* genome were incomplete, which made it impossible to determine their domain structure and use them in phylogenetic analysis. Therefore, in order to fill the gaps in available data, instead of incomplete sequences of *A. japonicus*, we used orthologous sequences from the previously sequenced and described transcriptome of the holothurian *Eupentacta fraudatrix* (D’yakonov & Baranova in D’yakonov, Baranova & Savel’eva, 1958) (GHCL00000000.2) [184]. Furthermore, in case of finding significant differences in the structure of any protein in the echinoderms under study, we additionally used data on other Echinodermata species in order to more accurately identify the domain structures using software. Thus, we used sequences from the genomes of the sea stars *Asterias rubens* (Linnaeus, 1758) (PRJNA626669) and *Acanthaster planci* (Linnaeus, 1758) (PRJNA397419), the sea urchins *Lytechinus pictus* (Verrill, 1867) (PRJNA889359) and *L. variegatus* (Lamarck, 1816) (PRJNA729485), and the holothurian *Holothuria leucospilota* (Brandt, 1835) (PRJNA747844).

Hereinafter, the accession numbers of the genomes and amino acid sequences from the NCBI database (https://www.ncbi.nlm.nih.gov, accessed on 20 April 2023) are shown in parentheses. The sequences used for the analysis are presented in Supplementary Files S1–S16. 

For phylogenetic analysis, in addition to data on echinoderms, we also used sequences of chordates (*Homo sapiens* (Linnaeus, 1767), *Ciona intestinalis* (Linnaeus, 1767), and *Branchiostoma belcheri* (Gray, 1847)) and hemichordates (*Saccoglossus kowalevskii* (Agassiz, 1873)). The trees were rooted on outgroup taxa represented by *Crassostrea gigas* (Thunberg, 1793) or *Nematostella vectensis* (Stephenson, 1935).

The phylogenetic trees were based on conserved regions of the putative amino acid sequences. The conserved regions were determined using the Gblock program. The construction was carried out by the RAxML-HPC BlackBox algorithm at the online service CIPRES (http://www.phylo.org, accessed on 20 April 2023). The trees were visualized using the iTOL v6.6 online tool (https://itol.embl.de; accessed on 20 April 2023). Domain structures of proteins were identified using the NCBI CD Search (https://www.ncbi.nlm.nih.gov/Structure/cdd/wrpsb.cgi, accessed on 20 April 2023) and Smart software (http://smart.embl-heidelberg.de/#, accessed on 20 April 2023). In addition, the SignalP-5.0 Server (http://www.cbs.dtu.dk/services/SignalP, accessed on 20 April 2023) and Phobius (https://phobius.sbc.su.se/index.html, accessed on 20 April 2023) were used to more accurately detect the presence of a signal peptide and transmembrane domains in amino acid sequences.

## 6. Conclusions

Echinoderms possess a vast set of genes encoding various ECM proteins. Accordingly, the connective tissue of these animals has a rather complex structure. Several groups of collagens exist that can potentially have different properties and perform a wide range of functions. In particular, fibril-forming collagens probably constitute the basis of ECM. Other types of collagens (FACITs and multiplexins), similarly to their homologs in vertebrates, can be involved in the network formation and in giving the connective tissue various properties. Collagens XXVI may probably play an important role in regulating the mechanical properties of connective tissue. The presence of the EMI domain allows them to interact with many ECM proteins. Multiplexins may be of certain importance for maintaining MCT properties, e.g., providing elasticity of ligaments. It is evident that such a complex ECM structure and the variety of intermolecular interactions determine the complexity of mechanisms changing the mechanical properties of connective tissue in echinoderms. These mechanisms probably depend not only on the number of cross-links between the molecules but also on the composition of ECM and the properties of its proteins. In this regard, more attention should be paid to the structure and functions of fibrillins in echinoderms. It is possible that the fibrillin microfibril scaffold, like that of vertebrates, forms a niche for regulatory factors and mechanosensation. Conducting a signal from the extracellular microenvironment to competent cells can be a part of the mechanisms of MCT mutability.

Various proteases play a major role in ECM remodeling. More in-depth studies of MCT properties and functions are needed to understand the transformation mechanisms. Furthermore, attention should be paid to various molecules that activate or inhibit proteases. Echinoderms have a wide range of proteins that can regulate the activity of proteases at various levels such as furin, RECK, and TIMPs. Tensilins apparently represent a separate group of holothurian proteins which probably have a specific function. They can be involved in the mechanisms of MCT transformation, acting as MCT stiffening factors or inhibitors of MMPs. However, their homologs/analogs in other echinoderms have not yet been identified.

## Data Availability

No new data were created or analyzed in this study. Data sharing is not applicable to this article.

## Supplementary Materials

The sequences used for the analysis can be downloaded at: https://www.mdpi.com/article/10.3390/md21070417/s1, File S1: Fibril forming collagens; File S2: FACITs; File S2: FACITs; File S3: Network collagens; File S4: Collagens 6_7_26_28; File S5: Multiplexins; File S6: Unknown collagens; File S7: Syndecans; File S8: Glypicans; File S9: Nidogens; File S10: Fibulins; File S11: Hemicentins; File S12: Thrombospondins; File S13: Fibrillins; File S14: Lysyl oxidases; File S15: Transglutaminases; File S16: TIMPs and tensilins; File S17: TIMPs and tensilins—deleted sequences from alingment.
